# Progression of Microstructural Degeneration in Progressive Supranuclear Palsy and Corticobasal Syndrome: A Longitudinal Diffusion Tensor Imaging Study

**DOI:** 10.1371/journal.pone.0157218

**Published:** 2016-06-16

**Authors:** Yu Zhang, Rudolph Walter, Peter Ng, Phi N. Luong, Shubir Dutt, Hilary Heuer, Julio C. Rojas-Rodriguez, Richard Tsai, Irene Litvan, Bradford C. Dickerson, Maria Carmela Tartaglia, Gil Rabinovici, Bruce L. Miller, Howard J. Rosen, Norbert Schuff, Adam L. Boxer

**Affiliations:** 1 Center for Imaging of Neurodegenerative Diseases, VA Medical Center San Francisco, San Francisco, CA, United States of America; 2 Department of Radiology and Biomedical Imaging, University of California San Francisco, San Francisco, CA, United States of America; 3 Department of Neurology, Memory and Aging Center, University of California San Francisco, San Francisco, CA, United States of America; 4 Department of Neurosciences, Movement Disorder Center, University of California San Diego, San Diego, CA, United States of America; 5 Department of Neurology, Massachusetts General Hospital, Boston, MA, United States of America; 6 University Health Network and Tanz Centre for Research in Neurodegenerative Disease, University of Toronto, Toronto, ON, Canada; University of Illinois at Chicago, UNITED STATES

## Abstract

Progressive supranuclear palsy (PSP) and corticobasal syndrome (CBS) are both 4 microtubule binding repeat tauopathy related disorders. Clinical trials need new biomarkers to assess the effectiveness of tau-directed therapies. This study investigated the regional distribution of longitudinal diffusion tensor imaging changes, measured by fractional anisotropy, radial and axial diffusivity over 6 months median interval, in 23 normal control subjects, 35 patients with PSP, and 25 patients with CBS. A mixed-effects framework was used to test longitudinal changes within and between groups. Correlations between changes in diffusion variables and clinical progression were also tested. The study found that over a 6 month period and compared to controls, the most prominent changes in PSP were up to 3±1% higher rates of FA reduction predominantly in superior cerebellar peduncles, and up to 18±6% higher rates of diffusivity increases in caudate nuclei. The most prominent changes in CBS compared to controls were up to 4±1% higher rates of anisotropy reduction and 18±6% higher rates of diffusivity increase in basal ganglia and widespread white matter regions. Compared to PSP, CBS was mainly associated with up to 3±1% greater rates of anisotropy reduction around the central sulci, and 11±3% greater rates of diffusivity increase in superior fronto-occipital fascicules. Rates of diffusivity increases in the superior cerebellar peduncle correlated with rates of ocular motor decline in PSP patients. This study demonstrated that longitudinal diffusion tensor imaging measurement is a promising surrogate marker of disease progression in PSP and CBS over a relatively short period.

## Introduction

Progressive supranuclear palsy (PSP) and corticobasal degeneration (CBD) are both sporadic atypical parkinsonian disorders associated with abnormal 4 microtubule binding domain (4R) tau protein accumulation in specific central nervous system neurons and glia, causing progressive disability and death [1–3]. PSP is pathologically characterized by tau lesions mainly in the basal ganglia and the brainstem [4], and clinically by postural instability and vertical supranuclear gaze palsy [5]. CBD in contrast is pathologically characterized by tau lesions in the frontoparietal and motor cortices [6], while the disorder can clinically show a variety of phenotypes. The most frequent presentation of CBD is with the corticobasal syndrome (CBS) characterized by unilateral dystonia, rigidity, myoclonus, parkinsonism, alien limb and ideomotor apraxia [7]. CBS can also be produced by pathology associated with other disorders such as Alzheimer’s disease [8]. There is emerging evidence that pure 4R tauopathies without co-occurrence of toxic amyloid plaques show faster brain atrophy than Alzheimer’s disease, which involves both tau and amyloid [9].

As new pharmacologic agents targeting tau accumulation are being developed to treat 4R tauopathies, there is an urgent need for powerful biomarkers that can accurately measure disease progression and assess the effectiveness of therapeutic interventions. Previous biomarker studies of disease progression in PSP and CBS using MRI have focused on measuring rates of regional gray matter atrophy [10–14]. Recently, there has been growing interests in also assessing microscopic white matter degeneration, such as demyelination or loss of axonal fiber bundles, using diffusion tensor imaging (DTI) [15]. A longitudinal DTI study [16] in PSP reported an increase in tissue water diffusivity in the putamen over time. However, the regional distribution of such microstructural changes beyond the putamen remains largely unknown. It is also unknown whether PSP and CBS each exhibit a characteristic pattern of regionally progressive brain tissue damage that might reflect their respective disease progression.

This multicenter study investigates the progression of regional microstructural degeneration in PSP and CBS using longitudinal DTI measurements. To the best of our knowledge, a longitudinal DTI study of the regional distribution and rates of progressive microstructural degeneration in PSP and CBS has not been reported before. The main goals are: first, to determine the pattern of regional microstructural changes in PSP and CBS as well as the degree to which the changes exceed those seen in normal aging, potentially providing an imaging marker of disease progression; second, to test the degree to which regional microstructural changes correlate with growing clinical disability in PSP and CBS.

## Materials and Methods

### Subjects

Participants were recruited as part of two neuroimaging initiatives: the Four Repeat Tauopathy Neuroimaging Initiative (4RTNI), which enrolled PSP and CBS patients, and the Neuroimaging Initiative for Frontotemporal Lobar Degeneration (FTLDNI), which enrolled healthy subjects. The study was approved by the Institutional Review Board of each participating site and all subjects or their legal guardians gave informed written consent. Both initiatives were managed by the University of California at San Francisco (UCSF) and followed the same principle study design and protocols for collecting clinical data and 3T Tesla MRI scans at three sites: UCSF, University of California at San Diego (UCSD) and University Health Network (UHN), University of Toronto. At UCSF, the MRI data were centrally checked for quality and processed. A total of 92 subjects had two sequential MRI scans with a six months median scan interval (range: 5 to 16 months). Data from six subjects (1 control, 4 PSP and 1 CBS) had to be excluded because of egregious imaging artifacts. Of the remaining 86 subjects, 35 met criteria for probable PSP [4], 25 for CBS (of those 23 were probable CBS [7], 2 had comorbid Alzheimer’s disease [17]), and 23 were cognitively normal control subjects. Autopsy confirmed diagnosis is available for 7 PSP and one CBS patients. A subset of CBS patients (16 out of 25) received amyloid assessments. Fourteen patients showed no evidence of elevated amyloid. Two CBS patients were amyloid positive, but were included in the analyses based on a supplemental study [18] that showed volumetric changes in these individuals were similar to their respective diagnostic groups. The population demographic and clinical characteristics are summarized in Table 1.

**Table 1:** Demographic and clinical characteristics.

Information	Control	PSP			CBS		Cross-sectional group differences (*p*)		Longitudinal changes (*p*)		
	Baseline	Baseline	Follow-up		Baseline	Follow-up	Patients vs. Control	PSP vs. CBS	PSP	CBS	PSP vs. CBS
**No. of MRI scans**	23	35			25		—		—	—	—
**Age at baseline MRI**	66.0 ± 7.3	70.0 ± 7.7			65.6 ± 6.9		*n*.*s*.		—	—	—
**Sex (% male)**	43	43			44		*n*.*s*.		—	—	—
**Mean MRI interval (ranges) (mo)**	7.8 (5–16)	6.4 (5–11)			7.0 ± 2.0 (5–14)		—		—	—	—
**Handedness**	21R	30R: 5L			24R: 1L		*n*.*s*.		—	—	—
**Side of motor symptoms**^**[**^^**1**^^]^*	—	13R: 11L: 9Sym			13R: 7L		—		—	—	—
Years of symptoms*	—	5.2 ± 4.3			4.5 ± 3.3		*n*.*s*.		—	—	—
**Clinical visit interval (mo)**	7.4 ± 2.8	6.5 ± 1.1			6.9 ± 2.1		—		—	—	—
**Amyloid**^**[**^^**2**^^**]**^ **(Neg:Pos:NA)**	—	—			14Neg:2Pos:9NA		—		—	—	—
**Autopsy confirmed subjects**	—	7			1		—		—	—	—
**L-dopa History (% On)**	—	48.6		51.4	56.0	48.0	—	—	—	—	—
**L-dopa at MRI (% On)**	—	37.1		30.6	40.0	36.0	—	—	—	—	—
**PSPRS total**^**[**^^**3**^^]^*	—	36.8 ± 13		39.8 ± 15	24.7 ± 9.2	32.2 ± 11	—	0.005^[^^b^^]^	0.007	<0.001	0.01^[^^c^^]^
**PSPRS-Bulbar**^**[**^^**4**^^**]**^	—	2.8 ± 1.5		3.1 ± 1.4	1.4 ± 1.0	2.2 ± 1.4	—	0.002^[^^b^^]^	*n*.*s*.	*n*.*s*.	*n*.*s*.
**PSPRS-Ocular Motor**^**[**^^**4**^^**]**^	—	7.1 ± 3.7		8.1 ± 3.9	1.6 ± 1.9	3.5 ± 3.6	—	<0.001^[^^b^^]^	0.02	0.003	*n*.*s*.
**PSPRS-Limb Motor**^**[**^^**4**^^**]**^	—	5.1 ± 2.7		5.2 ± 3.2	8.2 ± 3.6	9.6 ± 4.0	—	<0.001^[^^c^^]^	*n*.*s*.	0.01	0.02^[^^c^^]^
**PSPRS-Gait/Midline**^**[**^^**4**^^**]**^	—	10.0 ± 4.3		11.5 ± 5.1	5.2 ± 4.7	7.3 ± 4.9	—	0.002^[^^b^^]^	0.01	<0.001	*n*.*s*.
**UPDRS-III total**^**[**^^**5**^^]^*	—	29.1 ± 13		32.3 ± 14	26.8 ± 11	32.8 ± 12	—	*n*.*s*.	*n*.*s*.	0.001	*n*.*s*.
**CDR Box**^**[**^^**6**^^]^*	0.1 ± 0.5	4.1 ± 2.6		3.5 ± 2.7	3.2 ± 2.8	3.1 ± 3.6	0.001^[^^a^^]^	*n*.*s*.	*n*.*s*.	*n*.*s*.	*n*.*s*.
**MMSE**^**[**^^**7**^^]^*	29.2 ± 1.0	25.7 ± 4.0		25.2 ± 3.8	24.9 ± 4.2	24.0 ± 5.1	<0.001^[^^a^^]^	*n*.*s*.	*n*.*s*.	*n*.*s*.	*n*.*s*.
**MoCA total**^**[**^^**8**^^]^*	27.7 ± 2.1	22.1 ± 4.1		21.6 ± 3.9	19.8 ± 6.4	19.6 ± 6.9	<0.001^[^^a^^]^	*n*.*s*.	*n*.*s*.	*n*.*s*.	*n*.*s*.
**FAQ**^**[**^^**9**^^]^*	0.4 ± 0.5	13.8 ± 7.6		15.1 ± 7.7	10.9 ± 8.7	13.2 ± 9.0	<0.001^[^^a^^]^	*n*.*s*.	*n*.*s*.	*n*.*s*.	*n*.*s*.
**SEADL**^**[**^^**10**^^**]**^(%)*	100	59 ± 27		46 ± 26	58 ± 20	49 ± 24	<0.001^[^^a^^]^	*n*.*s*.	0.002	0.03	*n*.*s*.

^[1]^ The dominant side of motor symptoms was determined by an experienced neurologist (J.C.R.) from clinical data reviews.

^[2]^ Numbers of patients that assessed with amyloid based on either amyloid imaging or CSF. Neg = amyloid negative cases; Pos = amyloid positive cases; NA = subjects do not have available amyloid assessment.

^[3]^ PSPRS = PSP Rating Scale, range from 0 (best) to 100 (worst).

^[4]^ Four motor subscores from total PSPRS were analyzed: PSPRS-Bulbar, PSPRS-Ocular Motor, PSPRS-Limb Motor, and PSPRS-Gait/Midline.

^[5]^ UPDRS-III = Part-III (motor exams) of the Unified Parkinson's Disease Rating Scale, range from 0 (best) to 108 (worst).

^[6]^ CDR Box = Clinical Dementia Rating Sum of Boxes, range from 0 (best) to 18 (worst).

^[7]^ MMSE = Mini-Mental State Examination, range from 0 (worst) to 30 (best).

^[8]^ MoCA = Montreal Cognitive Assessment, range from 0 (worst) to 30 (best).

^[9]^ FAQ = Functional Activities Questionnaire, range from 0 (best) to 30 (worst).

^[10]^ SEADL = Schwab and England Activities of Daily Living, range from 0% (worst) to 100% (best).

* Subject numbers of missing information: Side of motor symptoms in 2 PSP, 5 CBS; Years of Symptom in 8 PSP and 2 CBS; PSPRS total in 5 PSP and 7 CBS; UPDRS-III total in 9 PSP and 9 CBS; CDR Box in 12 Controls, 3 PSP and 1 CBS; MMSE in 3 PSP and 4 CBS; MoCA in 1 Control, 12 PSP and 4 CBS; FAQ in 12 Controls, 10 PSP and 3 CBS; SEADL in 6 Controls, 6 PSP and 8 CBS subjects were missing.

^**[a]**^ Scores significantly worse in PSP compared to control and CBS compared to control.

^**[b]**^ Scores significantly worse in PSP compared to CBS.

^**[c]**^ Scores significantly worse in CBS compared to PSP.—indicates not applicable. *n*.*s*. indicates not significant. mo = month. R:L:Sym = Right:Left:Symmetric. Neg:Pos:NA = Positive:Negative:Not-tested.

All 73 subjects who were enrolled in this study had a comprehensive neurological examination at baseline and follow-up visit that included: the PSP Rating Scale (PSPRS) [19] and the Unified Parkinson's Disease Rating Scale (UPDRS) [20] for assessing motor disabilities; the Mini-Mental State Examination (MMSE) [21] and the Montreal Cognitive Assessment (MoCA) [22] for assessing global cognitive impairment; the Clinical Dementia Rating (CDR) box scales [23], the Schwab and England Activities of Daily Living (SEADL) [24], and Functional Activities Questionnaire (FAQ) [25] for measuring global living abilities.

### MRI acquisition

Three MRI types, T1-weighted, T2-weighted, and DTI were collected using Siemens (Siemens Healthcare Inc., USA) or GE (General Electric Healthcare, USA) 3T MRI machines. Acquisition parameters of the T1-weighted images, using a three-dimensional magnetization-prepared rapid gradient echo (3D-MPRAGE) scheme, were: TR/TE/TI = 2300/3/900ms, flip angle of 9°, sagittal orientation with 256×240×160 matrix size, 1 mm^3^ isotropic voxel resolution. Parameters of T2-weighted images with fluid-attenuated inversion recovery were: TR/TE/TI = 6000/390/2100ms and same resolution as MPRAGE. DTI was acquired using a standardized 2D single-shot EPI sequence, which was initially optimized at UCSF and then installed at the two other centers. The scan parameters are: with TR/TE = 9200/82ms, a 128×128 matrix in-plane and 44 contiguous slices, yielding 2.7 mm^3^ isotropic resolutions. Four images with no diffusion sensitization (b_0_ image) and 41 directional diffusion-weighted images (*b* = 1000s/mm^2^) were acquired using parallel imaging with twofold acceleration to reduce susceptibility distortions and signal loss. A standardized DTI protocol for diffusion time, gradient strength, and spatial resolution was used to reduce variability between scanners. In addition and when appropriate, statistical tests were performed with and without the addition of scan centers as co-variate to determine the degree to which scanner variations potentially biased results.

### Image processing and regions-of-interest extraction

**Fig 1:**
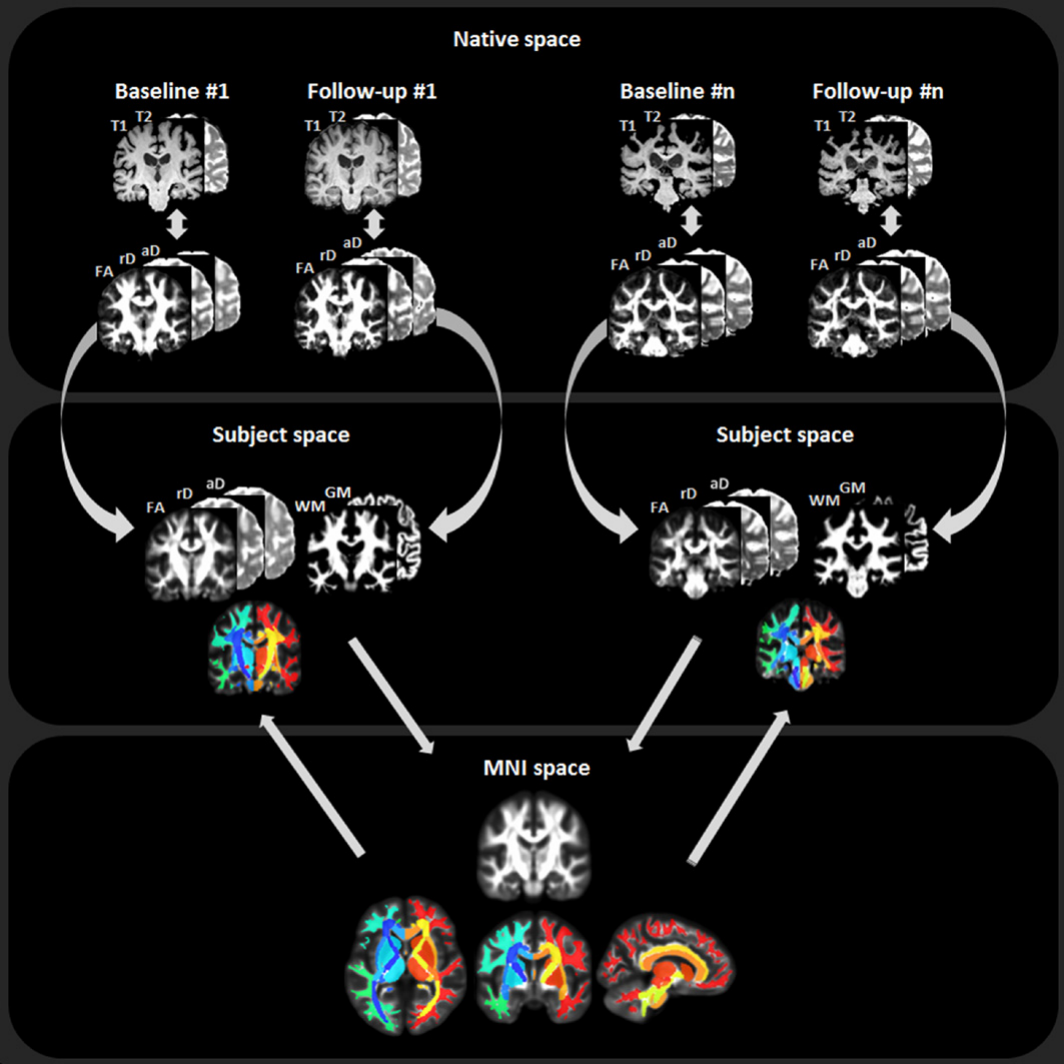
Illustration of the DTI processing pipeline for longitudinal analysis. FA = fractional Anisotropy; rD = radial diffusivity; aD = axial diffusivity; WM = white matter; GM = gray matter. See text for details of native, subject and MNI space definitions.

The structural MRI and DTI data were processed using a largely automated processing pipeline that is illustrated in Fig 1 and described also in a previous study [26]. Processing of DTI included corrections for motion, eddy-current effects and susceptibility distortions as well as voxelwise computation of fractional anisotropy (FA), radial diffusivity (rD), and axial diffusivity (aD). The DTI maps were then coregistered to the T1- weighted image via T2- weighted weighted image in the native space of each subject for each time point. To reduce bias in image coregistration toward a particular time point, a within-subject template was generated as reference by warping the corresponding baseline and follow-up images into the same space. Thereafter, an inter-subject registration of the within-subject templates across all subjects was performed via a nonlinear diffeomorphic registration (DARTEL) [27] that includes tissue segmentation, diffeomorphic warping, spatial normalization of the individual subject templates to the standard Montreal Neurological Institute (MNI) space [28] to accomplish anatomic labeling using the JHU-DTI-MNI (Type I WMPM) whole brain atlas [29]. Once in MNI space, all images are averaged to generate a population-specific template map. To further ensure that the population template matches well with the JHU-DTI-MNI atlas, an additional affine registration was performed between the JHU-DTI-MNI atlas and the population-specific template. Lastly, the JHU-DTI-MNI atlas was reversely transformed to each subject’s template to assign the anatomical labels onto the original images in their respective within-subject space. The anatomical labels were aggregated into 120 regions of interests (ROIs) that cover the entire white matter, including 42 white matter ROIs, 58 tract ROIs, and 20 basal ganglia and brainstem ROIs, for each baseline and follow-up image, respectively.

### Statistical analysis

All statistical tests were carried out using the R Project of Statistical Computing (http://www.r-project.org/). Regional variations in FA, rD, and aD changes were separately entered as dependent variables using linear mixed-effects regression model, in which a time-by-diagnosis interaction term was included to estimate a fixed group effect on the rate of DTI changes while variations in scan intervals across subjects were treated as random effects. Group differences were tested pairwise. Laterality of DTI changes were further tested by including a side-by-time interaction term. Following a similar design in our previous study [30], side was defined as the ipsilateral or contralateral hemisphere relative to the side of motor symptoms. For patients with a symmetric motor symptom and for control subjects, left and right hemispheric DTI measures were averaged. Correlations between regional DTI changes and rates of clinical symptom progression were evaluated using Pearson’s correlation coefficients. To control the type I error for multiple testing, the significance level of DTI measures was adjusted using the false discovery rate (FDR) [31]. All tests were two-tailed with a false discovery rate corrected significance level of *P*_*FDR*_<0.05.

## Results

### Demographics and neuropsychological findings

Demographic and clinical performance data at baseline and follow-up are summarized in Table 1. Patients and control subjects had similar age and gender distributions. At baseline, patients showed significant impairments in both motor and cognitive function compared to control subjects. PSP patients were more impaired than CBS at baseline based on total PSPRS and PSPRS subscores with the exception of the PSPRS Limb-motor subscore which showed CBS patients were more impaired. However, PSP and CBS patients had similar degrees of cognitive impairment and disease durations. Longitudinally, compared to control, both PSP and CBS patients showed significantly declined total PSPRS, PSPRS-Ocular Motor and PSPRS-Gait/Midline subscores, and SEADL scores. CBS patients further showed longitudinal decline in PSPRS Limb-motor subscore (*p* = 0.01) and total UPDRS score (*p* = 0.001) over the six months. In comparison between PSP and CBS, CBS patients declined clinically faster than PSP patients based on total PSPRS (*p* = 0.01) and PSPRS Limb-motor subscore (*p* = 0.02). Other clinical declines over time were not significant.

### Longitudinal DTI differences between groups

**Fig 2:**
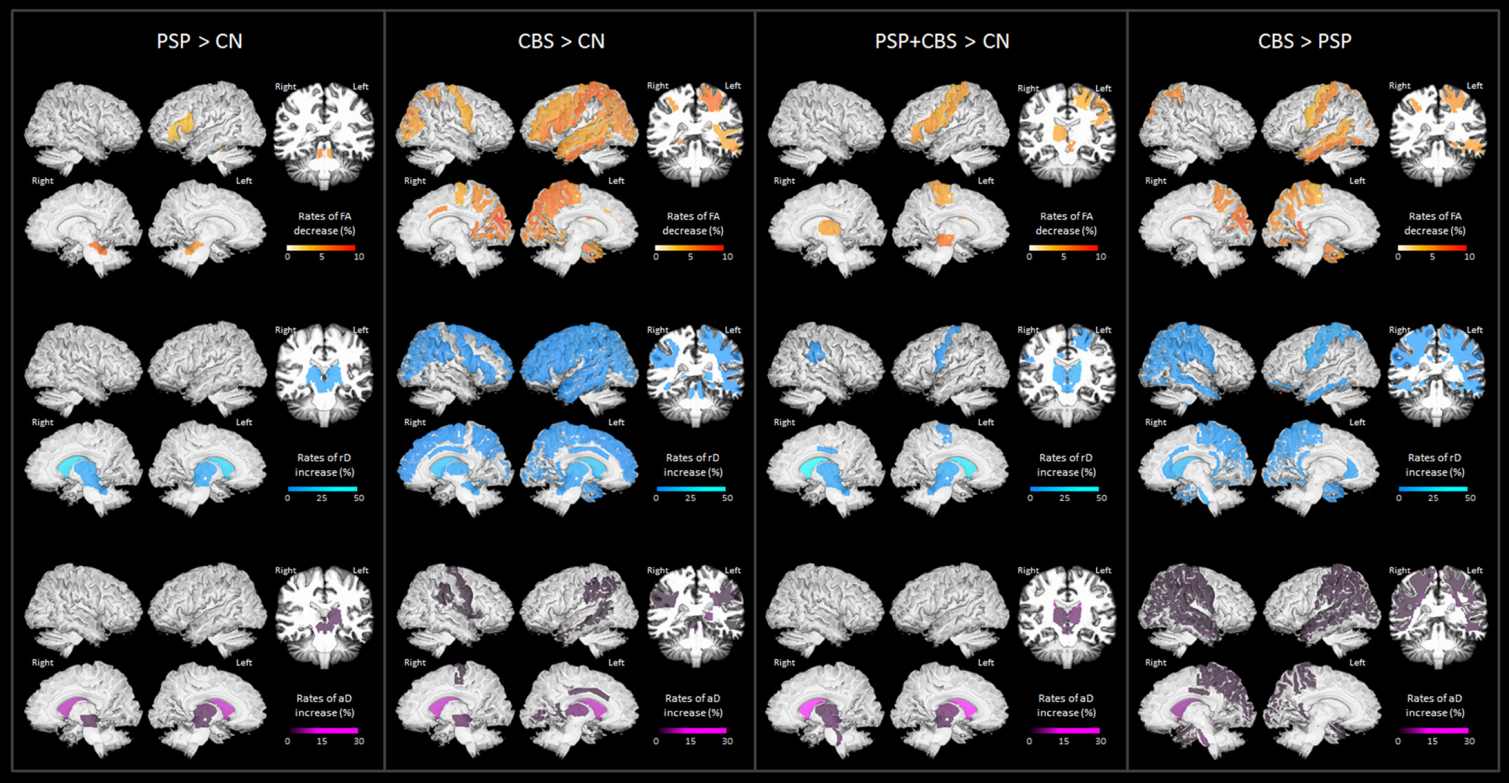
Surface-rendered brain maps of group differences in regional DTI rates. Row A: fractional anisotropy (FA); Row B: radial diffusivity (rD); Row C: axial diffusivity (aD). Color scales indicate rates of percent change from baseline within 6 months.

Regional patterns of group differences in FA, rD, and aD at baseline are illustrated in S1 Fig. The results are largely consistent with previous cross-sectional reports in literature [32–37]. Group differences in regional DTI changes over 6 months are illustrated in Fig 2 and S2 Fig. Tabulated summaries are given in S1 Table.

Compared to control subjects, PSP patients exhibited higher rates in FA reduction predominantly involving the superior cerebellar peduncles (left: 1.9±0.8% from baseline, *P*_*FDR*_ = 0.01, right: 2.6±1.2%, *P*_*FDR*_ = 0.02) and to some degree also the left inferior frontal white matter region. S2 Fig depicted individual trajectories of FA changes in the superior cerebellar peduncle, where PSP patients had specifically reduced FA at baseline and over time than control, whereas CBS patients had no significant baseline FA reduction than control, nor a significant FA reduction over time. PSP patients also had higher than normal rates in increased rD involving bilaterally the caudate nuclei (left: 15.8±5%, *P*_*FDR*_ = 0.004; right: 17.6±6%, *P*_*FDR*_ = 0.003) and to some degree the thalamus and midbrain. Furthermore, PSP patients had higher than normal rates of increased aD bilaterally in the caudate nuclei (left: 10.7±3%, *P*_*FDR*_ = 0.002; right: 9.6±3%, *P*_*FDR*_ = 0.004), and to some degree in the left thalamus and bilateral midbrain.

Compared to control subjects, CBS patients exhibited a diverse pattern of increased DTI abnormalities over 6 months. In detail, CBS patients showed higher than normal FA reduction rates between 2 to 4%±1% bilaterally in white matter regions including the frontal, parietal, occipital and temporal lobes. S2 Fig depicted individual trajectories of FA changes in the post-central white matter region, which showed the highest rates of FA reduction over time. In this region, CBS patients had specifically reduced FA at baseline and over time than control subjects, whereas PSP patients had intermediate FA reduction between CBS and control at baseline and had no greater FA reduction over time than control group. In addition to FA, CBS patients also had higher rates of rD increase that involved widespread supratentorial regions as well as the basal ganglia and brainstem. The bilateral caudate nuclei had the highest rate of rD increase (13±3%, *P*_*FDR*_<0.001). CBS patients further showed higher than normal rates in aD increase bilaterally in the caudate nuclei (11±2%, *P*_*FDR*_<0.001), as well as in the superior longitudinal fasciculus, mid-posterior part of the cingulum, and the supramarginal white matter regions.

Compared to PSP, CBS was associated with higher rates of up to 3±1% FA reduction over 6 months in the pre- and post-central, superior parieto-occipital as well as the temporal white matter regions, and higher rates of rD and aD increases in most of these regions, with the highest rates of rD and aD increases in the right superior fronto-occipital fasciculus (rD: 11±3%, *P*_*FDR*_<0.001; aD: 8.4±3%, *P*_*FDR*_ = 0.002). No brain region in PSP had higher DTI rates than CBS in any region.

Given the difficulty in pathologically separating CBD and PSP, an analysis was also performed with the patients pooled into a single group. Patients had around 2±1% (*P*_*FDR*_<0.05) higher than normal rates of FA reduction than control subjects in bilateral superior fronto-occipital fasciculus and bilateral midbrain was well as in left pre- and post-central white matter areas and right thalamus. Patients also had higher than normal rates of rD increase that reached up to 25±6%, (*P*_*FDR*_<0.001). The regions included bilaterally the caudate nucleus, the thalamus and the midbrain, and to some degree the mid-posterior cingulum as well as pre-central and supramarginal white matter areas. In addition, the patients had higher than normal rates of aD increase that reached up to 15±4% (*P*_*FDR*_<0.001) bilaterally in the caudate thalamus and midbrain.

Differences in DTI changes over time between the ipsilateral and contralateral side in patients were not significant.

### Clinical and imaging correlations

Correlations between DTI changes and clinical decline are summarized in Table 2. For correlations between the motor decline and DTI changes, in PSP, only rates of decline in PSPRS-Ocular subscores and rates of increased aD in the superior cerebellar peduncle were significantly correlated (*r* = 0.41, *P*_*FDR*_ = 0.03). In CBS, rates of decline in PSPRS-Limb subscores correlated with rates of all three DTI variables in the post-central white matter region (FA: *r* = -0.57, *P*_*FDR*_ = 0.01; rD: *r* = 0.53, *P*_*FDR*_ = 0.02; aD: *r* = 0.52, *P*_*FDR*_ = 0.03). Correlations between decline in total PSPRS (or total UPDRS) and DTI changes were not significant. For other clinical deficits (e.g. MMSE, MoCA, and SEADL), significant correlation were mainly observed in PSP group. In particular, decline in MoCA and SEADL correlated with rates of FA decline in the post-central region, and decline in MMSE correlated with changes over time of FA and rD in the mid-posterior cingulum.

**Table 2:** Pearson’s correlation co-efficient and *P*_*FDR*_ values showing associations between estimated rates of DTI and rates of clinical measures in selected ROIs.

Region of interest*	Measure	Statistics	PSPRS total		PSPRS Ocular Motor		PSPRS Limb Motor		UPDRS total		MMSE		MoCA		SEADL	
			In PSP	In CBS	In PSP	In CBS	In PSP	In CBS	In PSP	In CBS	In PSP	In CBS	In PSP	In CBS	In PSP	In CBS
Precentral	FA	Co-efficient	0.04	-0.08	0.08	0.10	0.18	-0.44	-0.23	-0.11	0.13	-0.10	-0.14	-0.11	-0.06	-0.11
WM		(*P*_*FDR*_)	(*n*.*s*.)	(*n*.*s*.)	(*n*.*s*.)	(*n*.*s*.)	(*n*.*s*.)	(*n*.*s*.)	(*n*.*s*.)	(*n*.*s*.)	(*n*.*s*.)	(*n*.*s*.)	(*n*.*s*.)	(*n*.*s*.)	(*n*.*s*.)	(*n*.*s*.)
	rD	Co-efficient	-0.10	0.10	-0.27	-0.12	-0.21	**0.60**	0.14	0.07	-0.02	0.19	0.19	0.26	0.04	-0.04
		(*P*_*FDR*_)	(*n*.*s*.)	(*n*.*s*.)	(*n*.*s*.)	(*n*.*s*.)	(*n*.*s*.)	**(<0.01)**	(*n*.*s*.)	(*n*.*s*.)	(*n*.*s*.)	(*n*.*s*.)	(*n*.*s*.)	(*n*.*s*.)	(*n*.*s*.)	(*n*.*s*.)
	aD	Co-efficient	-0.16	0.01	-0.34	-0.17	-0.19	0.42	-0.05	-0.02	0.07	0.27	0.15	0.35	0.07	-0.11
		(*P*_*FDR*_)	(*n*.*s*.)	(*n*.*s*.)	(*n*.*s*.)	(*n*.*s*.)	(*n*.*s*.)	(*n*.*s*.)	(*n*.*s*.)	(*n*.*s*.)	(*n*.*s*.)	(*n*.*s*.)	(*n*.*s*.)	(*n*.*s*.)	(*n*.*s*.)	(*n*.*s*.)
Postcentral	FA	Co-efficient	-0.35	-0.29	-0.11	-0.07	**-0.41**	**-0.57**	-0.04	-0.02	0.33	0.29	**0.40**	0.28	**0.37**	0.36
WM		(*P*_*FDR*_)	(*n*.*s*.)	(*n*.*s*.)	(*n*.*s*.)	(*n*.*s*.)	**(0.03)**	**(0.01)**	(*n*.*s*.)	(*n*.*s*.)	(*n*.*s*.)	(*n*.*s*.)	**(0.03)**	(*n*.*s*.)	**(0.05)**	(*n*.*s*.)
	rD	Co-efficient	-0.02	0.00	-0.14	-0.20	-0.16	**0.53**	0.18	-0.07	-0.13	0.18	0.03	0.09	0.03	-0.04
		(*P*_*FDR*_)	(*n*.*s*.)	(*n*.*s*.)	(*n*.*s*.)	(*n*.*s*.)	(*n*.*s*.)	**(0.02)**	(*n*.*s*.)	(*n*.*s*.)	(*n*.*s*.)	(*n*.*s*.)	(*n*.*s*.)	(*n*.*s*.)	(*n*.*s*.)	(*n*.*s*.)
	aD	Co-efficient	-0.02	0.12	-0.15	-0.18	-0.11	**0.51**	0.15	-0.15	0.04	0.07	0.11	0.02	0.02	-0.26
		(*P*_*FDR*_)	(*n*.*s*.)	(*n*.*s*.)	(*n*.*s*.)	(*n*.*s*.)	(*n*.*s*.)	**(0.03)**	(*n*.*s*.)	(*n*.*s*.)	(*n*.*s*.)	(*n*.*s*.)	(*n*.*s*.)	(*n*.*s*.)	(*n*.*s*.)	(*n*.*s*.)
Mid-	FA	Co-efficient	-0.07	0.03	0.06	0.14	0.10	-0.08	-0.06	0.01	**0.47**	0.07	0.26	0.25	-0.06	-0.16
posterior		(*P*_*FDR*_)	(*n*.*s*.)	(*n*.*s*.)	(*n*.*s*.)	(*n*.*s*.)	(*n*.*s*.)	(*n*.*s*.)	(*n*.*s*.)	(*n*.*s*.)	**(0.01)**	(*n*.*s*.)	(*n*.*s*.)	(*n*.*s*.)	(*n*.*s*.)	(*n*.*s*.)
Cingulum	rD	Co-efficient	-0.05	0.06	-0.17	-0.26	-0.28	0.11	-0.11	0.05	**-0.41**	-0.17	-0.20	-0.26	0.17	-0.08
		(*P*_*FDR*_)	(*n*.*s*.)	(*n*.*s*.)	(*n*.*s*.)	(*n*.*s*.)	(*n*.*s*.)	(*n*.*s*.)	(*n*.*s*.)	(*n*.*s*.)	**(0.02)**	(*n*.*s*.)	(*n*.*s*.)	(*n*.*s*.)	(*n*.*s*.)	(*n*.*s*.)
	aD	Co-efficient	-0.21	0.14	-0.22	-0.01	-0.27	0.04	-0.28	0.04	0.03	-0.08	0.09	0.13	0.18	-0.38
		(*P*_*FDR*_)	(*n*.*s*.)	(*n*.*s*.)	(*n*.*s*.)	(*n*.*s*.)	(*n*.*s*.)	(*n*.*s*.)	(*n*.*s*.)	(*n*.*s*.)	(*n*.*s*.)	(*n*.*s*.)	(*n*.*s*.)	(*n*.*s*.)	(*n*.*s*.)	(*n*.*s*.)
Superior	FA	Co-efficient	-0.30	-0.06	-0.20	-0.11	-0.11	0.18	-0.16	0.03	0.07	-0.02	0.17	-0.01	0.04	0.04
Cerebellar		(*P*_*FDR*_)	(*n*.*s*.)	(*n*.*s*.)	(*n*.*s*.)	(*n*.*s*.)	(*n*.*s*.)	(*n*.*s*.)	(*n*.*s*.)	(*n*.*s*.)	(*n*.*s*.)	(*n*.*s*.)	(*n*.*s*.)	(*n*.*s*.)	(*n*.*s*.)	(*n*.*s*.)
Peduncle	rD	Co-efficient	0.32	0.09	0.28	-0.18	0.05	0.06	0.14	-0.17	-0.05	-0.23	-0.19	-0.16	-0.04	-0.15
		(*P*_*FDR*_)	(*n*.*s*.)	(*n*.*s*.)	(*n*.*s*.)	(*n*.*s*.)	(*n*.*s*.)	(*n*.*s*.)	(*n*.*s*.)	(*n*.*s*.)	(*n*.*s*.)	(*n*.*s*.)	(*n*.*s*.)	(*n*.*s*.)	(*n*.*s*.)	(*n*.*s*.)
	aD	Co-efficient	0.25	0.26	**0.41**	-0.13	0.08	0.12	0.02	-0.27	0.08	**-0.56**	-0.32	-0.40	-0.22	-0.28
		(*P*_*FDR*_)	(*n*.*s*.)	(*n*.*s*.)	**(0.03)**	(*n*.*s*.)	(*n*.*s*.)	(*n*.*s*.)	(*n*.*s*.)	(*n*.*s*.)	(*n*.*s*.)	**(0.01)**	(*n*.*s*.)	(*n*.*s*.)	(*n*.*s*.)	(*n*.*s*.)
Thalamus	FA	Co-efficient	-0.24	0.00	-0.01	0.17	-0.07	-0.38	-0.19	-0.09	0.20	0.10	0.12	0.14	-0.02	-0.02
		(*P*_*FDR*_)	(*n*.*s*.)	(*n*.*s*.)	(*n*.*s*.)	(*n*.*s*.)	(*n*.*s*.)	(*n*.*s*.)	(*n*.*s*.)	(*n*.*s*.)	(*n*.*s*.)	(*n*.*s*.)	(*n*.*s*.)	(*n*.*s*.)	(*n*.*s*.)	(*n*.*s*.)
	rD	Co-efficient	0.36	0.13	0.07	-0.14	0.23	0.28	0.04	0.15	**-0.39**	-0.36	-0.35	-0.34	-0.22	-0.13
		(*P*_*FDR*_)	(*n*.*s*.)	(*n*.*s*.)	(*n*.*s*.)	(*n*.*s*.)	(*n*.*s*.)	(*n*.*s*.)	(*n*.*s*.)	(*n*.*s*.)	**(0.04)**	(*n*.*s*.)	(*n*.*s*.)	(*n*.*s*.)	(*n*.*s*.)	(*n*.*s*.)
	aD	Co-efficient	0.32	-0.02	0.14	-0.36	0.16	0.26	0.08	0.10	-0.13	-0.19	-0.18	-0.13	-0.22	-0.00
		(*P*_*FDR*_)	(*n*.*s*.)	(*n*.*s*.)	(*n*.*s*.)	(*n*.*s*.)	(*n*.*s*.)	(*n*.*s*.)	(*n*.*s*.)	(*n*.*s*.)	(*n*.*s*.)	(*n*.*s*.)	(*n*.*s*.)	(*n*.*s*.)	(*n*.*s*.)	(*n*.*s*.)

FA = fractional anisotropy; rD = radial diffusivity; aD = axial diffusivity; WM = white matter. **Bold:** Significant correlations between rates of abnormal DTI and rates of clinical dysfunctions in each patients’ group. *n*.*s*.: *P*_*FDR*_ ≥ 0.05.

*Regions are not listed if there is no significant correlation observed between rates of clinical changes (e.g. PSPRS-Bulbar, PSPRS-Gait/Midline, or FAQ) and rates of DTI changes.

## Discussion

The main novel result of this study is that PSP and CBS are each associated with a distinct pattern of longitudinal DTI changes in white matter relative to normal aging. These patterns are consistent with histopathological studies [38–40] of the distributions of tau lesions in each disease. Furthermore, the finding that CBS yielded greater DTI changes than PSP and control subjects is consistent with the view that CBS pathology has a predilection for white matter. It is also noteworthy that microstructural changes were detectable over a relatively short imaging interval of only six months on average, consistent with a rapid progression of primary tauopathies [14]. Taken together, longitudinal DTI is potentially a surrogate marker for PSP and CBS progression and potentially useful for assessing disease modifying interventions.

In PSP, the finding of progressive FA reduction particularly involving the superior cerebellar peduncle is consistent with histopathological reports of high vulnerability of this region to tau accumulation [38]. The superior cerebellar peduncle consists mainly of dentatorubrothalamic tracts with efferents from the dentate nucleus of the cerebellum that ascend to the ventrolateral thalamus through the superior cerebellar peduncles. Degeneration and activated microglia along this tract are thought to be a hallmark of PSP pathology [41,42]. The characteristic degeneration of the superior cerebellar peduncle in PSP has been demonstrated in a number of cross-sectional DTI studies [35,37,43,44]. Our longitudinal results expand on these findings in that the degeneration of the superior cerebellar peduncle further progressed in a short period from damage that was already detectable at baseline, and suggest that FA changes in this key region may mirror the progression of PSP pathology.

Other prominent regions with high rates of diffusivity changes in PSP included the caudate, thalamus and midbrain. These regions are consistent with neuropathological distributions of tau-positive astrocytic inclusions in the basal ganglia, and the thalamus [39]. However, as to the biological underpinning that are reflected in large rD and aD changes in absence of major FA changes is notoriously complicated [45]. More DTI studies augmented by autopsy are necessary to determine the underpinning of rD and aD changes in PSP.

The distribution of the rapid DTI changes over time in CBS than control and PSP subjects is largely consistent with histopathological findings of diffuse cerebral tau accumulation in this disease [40]. Unlike the histopathological features of PSP that are characterized by neuronal loss, gliosis, and abundant neurofibrillary tangles in the basal ganglia, midbrain and brainstem [2], the extensive accumulation of astrocytic plaques and tau-immunoreactive inclusions throughout the white matter is a striking feature of CBD [46]. Our results fit well with autopsy in CBD that report larger burden of astrocytic plaques and tau-immunoreactive inclusions throughout the white matter than those observed in PSP and Pick’s disease [40]. These results over all, suggest that progressive white matter degeneration is a prominent feature of CBS. In contrast to our reports of up to 4% FA reduction and 13% diffusivity increases per 6 months, previous structural MRI studies [13,14] have only reported up to 4%/year progressive regional gray matter atrophy in autopsy confirmed CBD. Whether the DTI changes, reflecting progressing microstructural degeneration, precede the macroscopic atrophy remains to be determined in future studies that use structural MRI and DTI together.

Our findings from evaluating DTI data of PSP and CBS together versus evaluating them separately are worth a discussion in the context of difficulties mapping the clinical features of PSP and CBS to a common pathology [47]. Based on using PSP and CBS data together, the finding of progressive regional microstructural degeneration in primarily motor related white matter regions suggests that the conditions share a neuropathological spectrum beyond the diverging pattern of progressive white matter degenerations that differentiates them. Arguably it is important to recognize a patient’s individual need for a clear diagnosis of either CBS or PSP and a differential management plan despite the possibility that the two clinical phenotypes have overlapping pathological underpinnings.

We found that aD changes in the superior cerebellar peduncle best correlated with decline in PSPRS-Ocular motor subscores, although this correlation was significant only for aD, but weak for FA and rD. The lack of statistic powers of this correlation may due to short follow-up time, small sample size, or relatively later stage of the disease. However, the potential role of DTI changes of the superior cerebellar peduncle as marker of PSP progression should not be neglected, given the knowledge that selective damage to the superior cerebellar tracts contribute to the gaze palsy [48].

The dominance of increased regional rD rates as compared to regional FA rates seems a prominent feature in CBS and PSP. Although the biological underpinning of various DTI measures is notoriously difficult, increased rD is generally thought to indicate demyelination [49], whereas decreased FA has been associated with a variety of microstructural alterations, including loss or disruption of both axons, loss of degradation of myelin sheets, glial cells infiltration. We therefore conclude that a major component of white matter degradation in CBS and PSP is demyelination. DTI findings from animal studies suggest axonal damage leads to aD decrease in early axonal injury and aD increase in later stage of the damage [50]. Human cerebral studies showed aD increase in neuropathologies. We therefore cautiously interpret the finding of prominent regional aD increase in PSP and CBS as indication for axonal damage.

A limitation of the study is that diagnosis was based on clinical symptoms without autopsy confirmation in the majority of cases. While the clinical criteria are relatively accurate for identifying PSP pathology, they are known to lack specificity for CBD pathology [7]. Comorbid tau conditions in CBS patients, such as Alzheimer’s disease, might have biased the findings in CBS. In this study, absence of Alzheimer’s disease was established based on negative results from amyloid imaging or CSF analysis in the majority (88%) of CBS patients. However, other tau related conditions that confound pathologies of CBD couldn’t be fully excluded from the study. It is worthwhile to note that the finding of abnormal regional DTI values in CBS prevailed regardless whether the analysis was limited to the negative brain amyloid cases or also included the cases with unknown amyloid status. The result suggests that amyloid prevalence unlikely plays a major role in the abnormal DTI pattern in CBS. However, additional studies involving validated positive amyloid cases will be necessary to come to a more firm conclusion about the role of amyloid in CBS. Eventually, neuropathological confirmation of CBD is warranted to interpret the findings in relation to CBD pathology. Another limitation is that, although there was no evidence of bias from MRI machine variations across the three centers based on patient data (88% of DTI was collected using one machine at UCSF), the exclusion of potential bias in comparisons between DTI data from patients and control subjects is not conclusive, because all controls were scanned on one machine. The assumption has been made that MRI machine variations are not group specific.

## Supporting Information

S1 Fig **Patterns of baseline group difference in FA (row A), rD (row B), and aD (row C)**.
(TIF)

S2 Fig **Individual trajectories of FA changes in A: the superior cerebellar peduncle, and B: the post-central white matter region of the control, PSP, and CBS groups**. Thick solid lines represent the mean change in each respective group.
(TIF)

S1 Table Estimated rates (percentage per 6 months) of FA, rD and aD changes within and between groups. (DOCX)
