# Supplementary figures and images for: Progression of Microstructural Degeneration in Progressive Supranuclear Palsy and Corticobasal Syndrome: A Longitudinal Diffusion Tensor Imaging Study
Source: PLoS One. 2016 Jun 16;11(6):e0157218. doi: 10.1371/journal.pone.0157218 (PMC4911077; doi:10.1371/journal.pone.0157218)

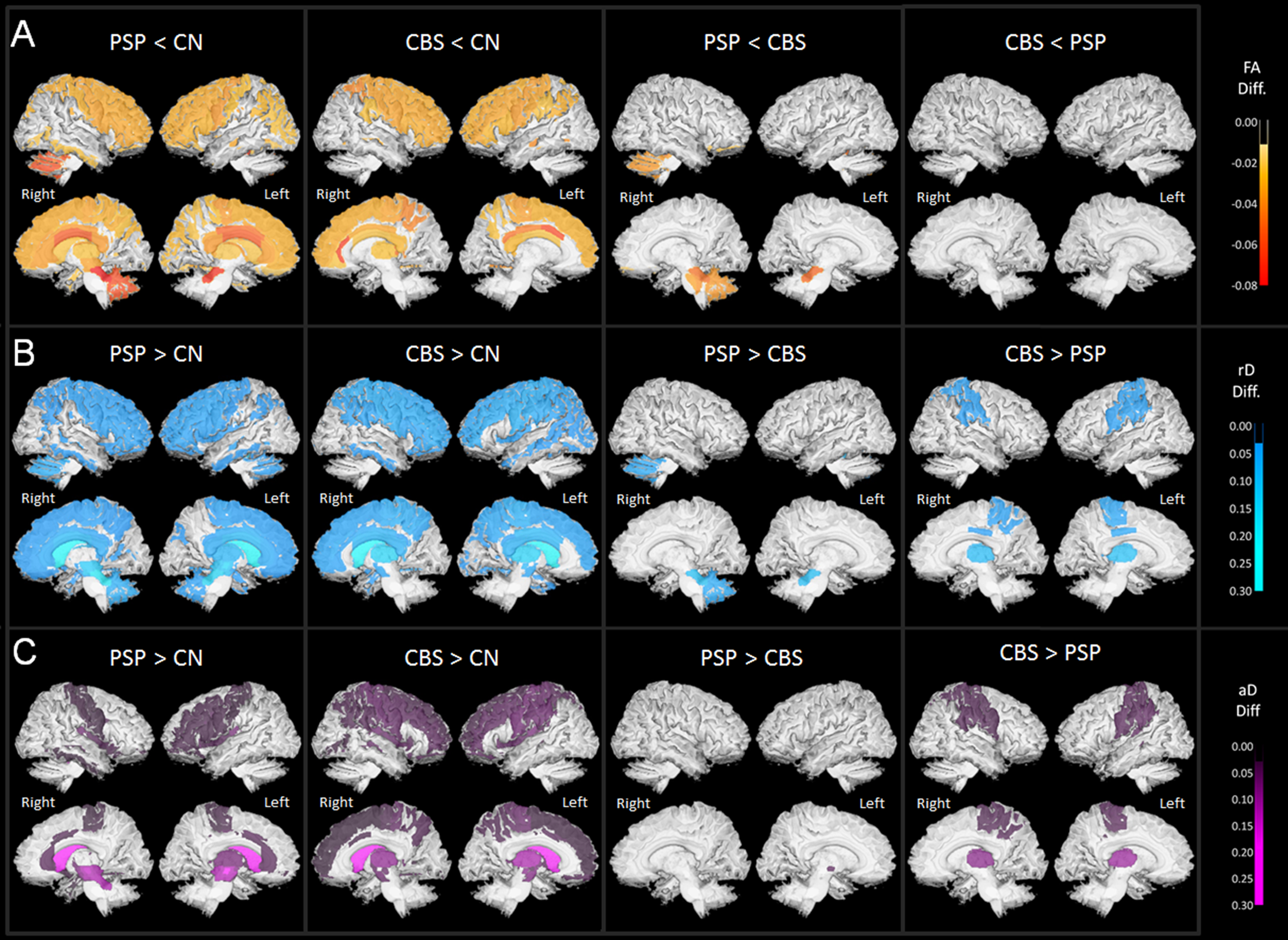

Supplement: S1 Fig — Patterns of baseline group difference in FA (row A), rD (row B), and aD (row C). (TIF) [file pone.0157218.s001.tif]

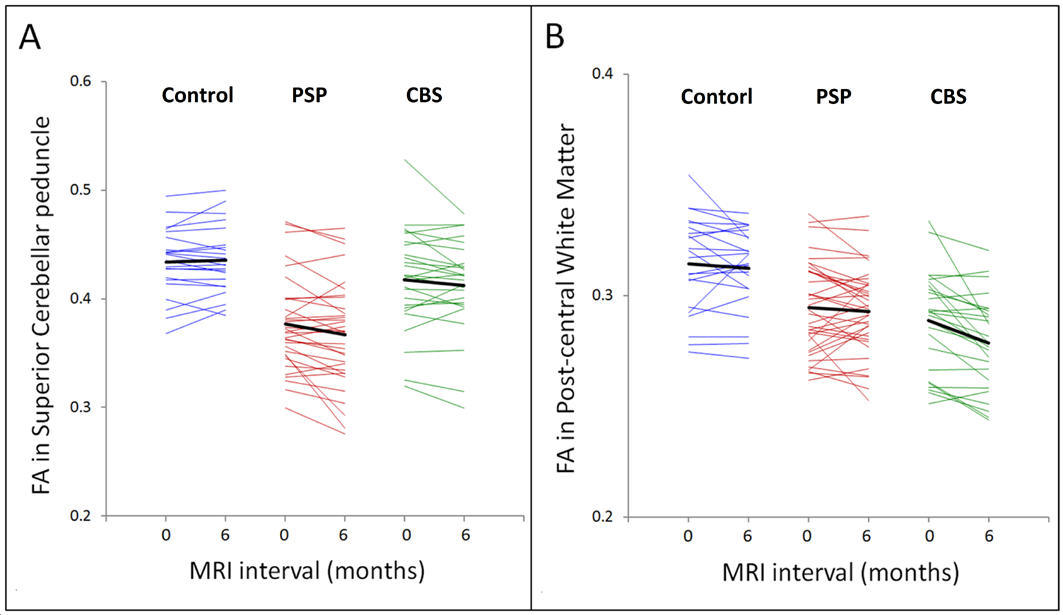

Supplement: S2 Fig — Individual trajectories of FA changes in A: the superior cerebellar peduncle, and B: the post-central white matter region of the control, PSP, and CBS groups. Thick solid lines represent the mean change in each respective group. (TIF) [file pone.0157218.s002.tif]
