# Supplementary material for: Progression of Microstructural Degeneration in Progressive Supranuclear Palsy and Corticobasal Syndrome: A Longitudinal Diffusion Tensor Imaging Study
Source: PLoS One. 2016 Jun 16;11(6):e0157218. doi: 10.1371/journal.pone.0157218 (PMC4911077; doi:10.1371/journal.pone.0157218)
Supplement: S1 Table — (DOCX) [file pone.0157218.s003.docx]

**S1 Table.** Estimated rates (percentage per 6 months) of FA, rD and aD changes within and between groups.

|  |  |  | **CN** | **PSP** | **CBS** | **PSP+CBS *vs.* CN** | | **PSP *vs.* CN** | | **CBS *vs.* CN** | | **CBS *vs.* PSP** | |
| --- | --- | --- | --- | --- | --- | --- | --- | --- | --- | --- | --- | --- | --- |
| Region of Interest* | Hemi-sphere | Measure | % Est. rate (S.E.) | % Est. rate (S.E.) | % Est. rate (S.E.) | % Est. rate (S.E.) | *P_FDR_* | % Est. rate (S.E.) | *P_FDR_* | % Est. rate (S.E.) | *P_FDR_* | % Est. rate (S.E.) | *P_FDR_* |
| Lateral fronto-orbital WM | Left | FA | -0.3 (0.5) | -0.2 (0.6) | -1.6 (0.6) | -0.9 (0.7) | *n.s.* | -0.2 (0.8) | *n.s.* | **-1.9 (0.8)** | **0.04** | -1.3 (0.8) | *n.s.* |
|  |  | rD | 1.1 (0.5) | 0.5 (0.7) | 1.5 (0.7) | -0.1 (0.8) | *n.s.* | -0.7 (0.9) | *n.s.* | 0.4 (0.8) | *n.s.* | 1.0 (0.9) | *n.s.* |
|  |  | aD | 1.2 (0.5) | 0.2 (0.5) | 0.6 (0.6) | -0.8 (0.7) | *n.s.* | -1.0 (0.8) | *n.s.* | 0.5 (0.7) | *n.s.* | 0.3 (0.7) | *n.s.* |
|  | Right | FA | -0.9 (0.6) | 0.4 (0.5) | -0.7 (0.5) | 0.4 (0.6) | *n.s.* | 1.1 (0.7) | *n.s.* | -0.3 (0.7) | *n.s.* | -1.3 (0.7) | *n.s.* |
|  |  | rD | 0.5 (0.5) | 0.2 (0.6) | 0.8 (0.6) | -0.0 (0.7) | *n.s.* | -0.3 (0.8) | *n.s.* | 0.3 (0.7) | *n.s.* | 0.5 (0.8) | *n.s.* |
|  |  | aD | 0.2 (0.4) | 0.7 (0.5) | 0.5 (0.5) | -0.4 (0.6) | *n.s.* | 0.5 (0.7) | *n.s.* | -0.3 (0.7) | *n.s.* | -0.2 (0.7) | *n.s.* |
| Inferior frontal WM | Left | FA | -0.1 (0.4) | -0.9 (0.4) | -2.2 (0.5) | **-1.6 (0.5)** | **0.001** | **-1.1 (0.5)** | **0.02** | **-2.5 (0.6)** | **<0.001** | -1.3 (0.6) | *n.s.* |
|  |  | rD | 0.5 (0.4) | 0.7 (0.6) | 2.0 (0.6) | 1.2 (0.7) | *n.s.* | 0.3 (0.7) | *n.s.* | **1.7 (0.6)** | **0.02** | 1.6 (0.8) | *n.s.* |
|  |  | aD | 0.5 (0.3) | -0.0 (0.4) | 0.6 (0.4) | -0.1 (0.5) | *n.s.* | -0.4 (0.5) | *n.s.* | 0.2 (0.5) | *n.s.* | 0.6 (0.5) | *n.s.* |
|  | Right | FA | -1.0 (0.5) | -1.2 (0.5) | -1.7 (0.5) | -0.9 (0.6) | *n.s.* | -0.7 (0.7) | *n.s.* | -1.4 (0.7) | *n.s.* | -0.5 (0.7) | *n.s.* |
|  |  | rD | 0.7 (0.4) | 1.3 (0.6) | 2.3 (0.6) | 1.2 (0.7) | *n.s.* | 0.7 (0.7) | *n.s.* | **1.6 (0.6)** | **0.03** | 1.1 (0.8) | *n.s.* |
|  |  | aD | 0.3 (0.4) | 0.4 (0.3) | 1.0 (0.5) | 0.5 (0.5) | *n.s.* | 0.2 (0.5) | *n.s.* | 0.8 (0.6) | *n.s.* | 0.6 (0.5) | *n.s.* |
| Middle frontal WM | Left | FA | -0.7 (0.4) | -1.2 (0.5) | -1.9 (0.6) | -1.2 (0.6) | *n.s.* | -0.8 (0.7) | *n.s.* | **-1.7 (0.7)** | **0.02** | -0.7 (0.8) | *n.s.* |
|  |  | rD | 0.9 (0.3) | 0.9 (0.7) | 2.0 (0.5) | 0.7 (0.7) | *n.s.* | 0.1 (0.8) | *n.s.* | **1.2 (0.5)** | **0.04** | 1.2 (0.8) | *n.s.* |
|  |  | aD | 0.7 (0.3) | 0.2 (0.4) | 0.9 (0.4) | -0.1 (0.5) | *n.s.* | -0.4 (0.6) | *n.s.* | 0.3 (0.4) | *n.s.* | 0.7 (0.5) | *n.s.* |
|  | Right | FA | -0.8 (0.5) | -0.8 (0.5) | -1.6 (0.6) | -0.7 (0.6) | *n.s.* | -0.4 (0.7) | *n.s.* | -1.1 (0.8) | *n.s.* | -0.6 (0.8) | *n.s.* |
|  |  | rD | 0.6 (0.4) | 1.4 (0.7) | 1.8 (0.6) | 1.1 (0.7) | *n.s.* | 0.8 (0.9) | *n.s.* | 1.2 (0.6) | *n.s.* | 0.6 (0.9) | *n.s.* |
|  |  | aD | 0.2 (0.3) | 0.6 (0.4) | 0.9 (0.4) | 0.6 (0.5) | *n.s.* | 0.5 (0.6) | *n.s.* | 0.7 (0.5) | *n.s.* | 0.3 (0.6) | *n.s.* |
| Superior frontal WM | Left | FA | -1.1 (0.5) | -1.3 (0.5) | -1.7 (0.5) | -0.8 (0.6) | *n.s.* | -0.5 (0.7) | *n.s.* | -1.1 (0.7) | *n.s.* | -0.6 (0.7) | *n.s.* |
|  |  | rD | 0.6 (0.3) | 1.1 (0.7) | 1.8 (0.5) | 1.0 (0.6) | *n.s.* | 0.5 (0.8) | *n.s.* | **1.3 (0.5)** | **0.006** | 0.8 (0.8) | *n.s.* |
|  |  | aD | 0.1 (0.3) | 0.1 (0.4) | 0.5 (0.3) | 0.2 (0.4) | *n.s.* | 0.0 (0.5) | *n.s.* | 0.4 (0.4) | *n.s.* | 0.4 (0.5) | *n.s.* |
|  | Right | FA | -0.8 (0.5) | -0.9 (0.5) | -1.3 (0.5) | -0.7 (0.6) | *n.s.* | -0.4 (0.7) | *n.s.* | -1.0 (0.6) | *n.s.* | -0.6 (0.7) | *n.s.* |
|  |  | rD | 0.7 (0.4) | 0.8 (0.6) | 2.0 (0.5) | 0.8 (0.7) | *n.s.* | 0.2 (0.8) | *n.s.* | **1.3 (0.6)** | **0.04** | 1.2 (0.8) | *n.s.* |
|  |  | aD | 0.3 (0.3) | -0.0 (0.4) | 0.7 (0.3) | 0.1 (0.4) | *n.s.* | -0.3 (0.5) | *n.s.* | 0.4 (0.4) | *n.s.* | 0.6 (0.5) | *n.s.* |
| Precentral WM | Left | FA | -0.3 (0.6) | -0.7 (0.5) | -1.6 (0.4) | **-1.3 (0.6)** | **0.01** | -0.7 (0.7) | *n.s.* | **-2.0 (0.6)** | **0.001** | **-1.3 (0.6)** | **0.03** |
|  |  | rD | 0.4 (0.5) | 0.6 (0.6) | 4.1 (0.8) | **1.9 (0.8)** | **0.02** | 0.5 (0.8) | *n.s.* | **3.1 (0.7)** | **<0.001** | **2.8 (0.9)** | **0.002** |
|  |  | aD | 0.2 (0.4) | 0.1 (0.4) | 1.2 (0.4) | 0.5 (0.5) | *n.s.* | -0.0 (0.5) | *n.s.* | 1.1 (0.5) | *n.s.* | **1.1 (0.5)** | **0.04** |
|  | Right | FA | -0.9 (0.5) | -0.8 (0.5) | -1.8 (0.4) | -0.8 (0.5) | *n.s.* | -0.2 (0.6) | *n.s.* | **-1.5 (0.6)** | **0.004** | -1.3 (0.7) | *n.s.* |
|  |  | rD | 0.5 (0.4) | 0.7 (0.6) | 3.5 (0.8) | 1.6 (0.8) | *n.s.* | 0.3 (0.8) | *n.s.* | **2.7 (0.8)** | **0.001** | **2.7 (0.9)** | **0.007** |
|  |  | aD | 0.0 (0.4) | 0.0 (0.4) | 1.3 (0.4) | 0.7 (0.5) | *n.s.* | 0.1 (0.5) | *n.s.* | **1.3 (0.5)** | **0.03** | **1.3 (0.6)** | **0.03** |
| Postcentral WM | Left | FA | 0.1 (0.6) | -0.4 (0.6) | -2.1 (0.6) | **-1.6 (0.7)** | **0.01** | -0.6 (0.8) | *n.s.* | **-2.8 (0.8)** | **<0.001** | **-2.1 (0.8)** | **0.02** |
|  |  | rD | 0.3 (0.4) | 0.2 (0.7) | 3.5 (0.8) | 1.6 (0.8) | *n.s.* | 0.1 (0.8) | *n.s.* | **2.8 (0.8)** | **0.001** | **3.0 (1.0)** | **0.003** |
|  |  | aD | 0.3 (0.4) | -0.1 (0.4) | 1.4 (0.4) | 0.5 (0.5) | *n.s.* | -0.3 (0.6) | *n.s.* | 1.1 (0.6) | *n.s.* | **1.4 (0.6)** | **0.02** |
|  | Right | FA | -1.9 (0.8) | -0.4 (0.6) | -2.0 (0.6) | -0.1 (0.7) | *n.s.* | 0.8 (0.9) | *n.s.* | -1.1 (0.9) | *n.s.* | -1.8 (0.8) | *n.s.* |
|  |  | rD | 1.2 (0.4) | 0.0 (0.6) | 2.7 (0.7) | 0.2 (0.8) | *n.s.* | -1.2 (0.8) | *n.s.* | 1.5 (0.7) | *n.s.* | **2.7 (0.9)** | **0.004** |
|  |  | aD | 0.5 (0.3) | -0.3 (0.4) | 1.3 (0.4) | 0.0 (0.5) | *n.s.* | -0.8 (0.5) | *n.s.* | 0.8 (0.5) | *n.s.* | **1.5 (0.5)** | **0.007** |
| Superior parietal WM | Left | FA | -0.3 (0.7) | -0.3 (0.6) | -2.4 (0.7) | -1.4 (0.8) | *n.s.* | -0.3 (0.8) | *n.s.* | **-2.8 (0.9)** | **0.007** | **-2.1 (0.9)** | **0.05** |
|  |  | rD | 0.5 (0.5) | 0.1 (0.7) | 3.4 (0.9) | 1.6 (0.9) | *n.s.* | -0.1 (0.9) | *n.s.* | **2.9 (0.9)** | **0.004** | **3.6 (1.1)** | **0.002** |
|  |  | aD | 0.3 (0.4) | -0.3 (0.4) | 1.4 (0.4) | 0.3 (0.5) | *n.s.* | -0.5 (0.5) | *n.s.* | **1.1 (0.5)** | **0.04** | **1.6 (0.6)** | **0.003** |
|  | Right | FA | -1.3 (0.6) | -0.3 (0.5) | -2.6 (0.7) | -0.7 (0.7) | *n.s.* | 0.3 (0.8) | *n.s.* | **-2.1 (0.9)** | **0.03** | **-2.1 (0.9)** | **0.04** |
|  |  | rD | 0.9 (0.4) | -0.0 (0.6) | 2.9 (0.7) | 0.7 (0.8) | *n.s.* | -0.9 (0.7) | *n.s.* | **2.0 (0.7)** | **0.009** | **3.1 (0.9)** | **0.001** |
|  |  | aD | 0.4 (0.3) | -0.3 (0.4) | 1.0 (0.4) | -0.0 (0.5) | *n.s.* | -0.7 (0.5) | *n.s.* | 0.6 (0.5) | *n.s.* | **1.3 (0.5)** | **0.01** |
| Precuneus WM | Left | FA | -0.8 (0.5) | -0.2 (0.5) | -2.4 (0.7) | -0.9 (0.7) | *n.s.* | 0.2 (0.7) | *n.s.* | **-2.3 (0.8)** | **0.03** | **-2.0 (0.8)** | **0.02** |
|  |  | rD | 0.6 (0.4) | -0.2 (0.7) | 2.3 (0.6) | 0.6 (0.8) | *n.s.* | -0.8 (0.8) | *n.s.* | **1.8 (0.7)** | **0.04** | **2.7 (0.9)** | **0.008** |
|  |  | aD | 0.5 (0.4) | -0.3 (0.5) | -1.3 (0.5) | 0.1 (0.6) | *n.s.* | -0.7 (0.7) | *n.s.* | 0.9 (0.6) | *n.s.* | **1.5 (0.7)** | **0.04** |
|  | Right | FA | -2.0 (0.7) | -0.2 (0.6) | -3.3 (1.1) | -0.5 (0.9) | *n.s.* | 0.9 (0.9) | *n.s.* | -2.3 (1.3) | *n.s.* | -2.8 (1.1) | *n.s.* |
|  |  | rD | 0.8 (0.4) | -0.5 (0.6) | 2.8 (0.8) | 0.6 (0.8) | *n.s.* | -1.2 (0.8) | *n.s.* | **2.2 (0.9)** | **0.02** | **3.4 (0.9)** | **0.001** |
|  |  | aD | 0.2 (0.4) | -0.6 (0.4) | 1.5 (0.6) | 0.3 (0.6) | *n.s.* | -0.8 (0.6) | *n.s.* | 1.4 (0.7) | *n.s.* | **2.1 (0.7)** | **0.006** |
| Supra-marginal WM | Left | FA | -0.2 (0.6) | -1.1 (0.7) | -1.2 (0.5) | -1.0 (0.7) | *n.s.* | -1.0 (0.9) | *n.s.* | -1.1 (0.7) | *n.s.* | -0.0 (0.8) | *n.s.* |
|  |  | rD | 0.4 (0.5) | 0.7 (0.7) | 2.2 (0.4) | **1.4 (0.7)** | **0.05** | 0.6 (0.8) | *n.s.* | **2.1 (0.6)** | **0.001** | **1.8 (0.7)** | **0.03** |
|  |  | aD | 0.3 (0.4) | 0.2 (0.5) | 1.9 (0.5) | 1.0 (0.6) | *n.s.* | 0.1 (0.7) | *n.s.* | **1.7 (0.6)** | **0.005** | **1.8 (0.6)** | **0.007** |
|  | Right | FA | -0.4 (0.5) | -1.2 (0.6) | -1.2 (0.5) | -0.9 (0.6) | *n.s.* | -0.8 (0.7) | *n.s.* | -1.0 (0.6) | *n.s.* | -0.2 (0.8) | *n.s.* |
|  |  | rD | 0.3 (0.3) | 0.8 (0.4) | 2.0 (0.5) | **1.2 (0.5)** | **0.04** | 0.5 (0.5) | *n.s.* | **1.8 (0.6)** | **0.002** | **1.4 (0.7)** | **0.02** |
|  |  | aD | 0.2 (0.3) | 0.0 (0.4) | 1.4 (0.5) | 0.7 (0.5) | *n.s.* | -0.0 (0.5) | *n.s.* | **1.3 (0.5)** | **0.008** | **1.4 (0.6)** | **0.003** |
| Angular WM | Left | FA | -0.3 (0.5) | -0.2 (0.7) | -2.4 (0.7) | -0.9 (0.7) | *n.s.* | 0.1 (0.8) | *n.s.* | **-2.2 (0.8)** | **0.02** | -2.2 (0.9) | *n.s.* |
|  |  | rD | 0.6 (0.5) | 0.4 (0.7) | 3.1 (0.7) | 1.4 (0.8) | *n.s.* | -0.1 (0.9) | *n.s.* | **2.6 (0.9)** | **0.004** | **2.9 (0.9)** | **0.004** |
|  |  | aD | 0.4 (0.4) | 0.2 (0.4) | 1.8 (0.5) | 0.8 (0.6) | *n.s.* | -0.0 (0.6) | *n.s.* | **1.5 (0.7)** | **0.02** | **1.6 (0.7)** | **0.01** |
|  | Right | FA | -0.8 (0.6) | -0.4 (0.5) | -2.3 (0.7) | -0.9 (0.7) | *n.s.* | -0.0 (0.7) | *n.s.* | **-2.1 (0.9)** | **0.04** | -1.8 (0.9) | *n.s.* |
|  |  | rD | 0.6 (0.3) | -0.1 (0.5) | 2.6 (0.6) | 0.8 (0.6) | *n.s.* | -0.6 (0.6) | *n.s.* | **2.0 (0.7)** | **0.004** | **2.8 (0.7)** | **0.001** |
|  |  | aD | 0.3 (0.3) | -0.3 (0.3) | 1.0 (0.4) | 0.1 (0.4) | *n.s.* | -0.6 (0.4) | *n.s.* | 0.7 (0.5) | *n.s.* | **1.3 (0.5)** | **0.007** |
| Superior occipital WM | Left | FA | -0.7 (0.8) | -0.6 (0.8) | -2.3 (0.7) | -1.3 (0.9) | *n.s.* | -0.3 (1.1) | *n.s.* | **-2.7 (1.0)** | **0.02** | -2.1 (1.0) | *n.s.* |
|  |  | rD | 0.5 (0.7) | 0.2 (0.9) | 3.4 (1.1) | 1.4 (1.1) | *n.s.* | -0.2 (1.1) | *n.s.* | **2.7 (1.2)** | **0.04** | **3.4 (1.4)** | **0.02** |
|  |  | aD | 0.3 (0.5) | -0.3 (0.4) | 1.2 (0.6) | 0.2 (0.6) | *n.s.* | -0.5 (0.6) | *n.s.* | 0.9 (0.7) | *n.s.* | 1.4 (0.7) | *n.s.* |
|  | Right | FA | -1.1 (0.9) | -0.0 (0.7) | -2.4 (0.7) | -0.9 (0.9) | *n.s.* | 0.4 (1.1) | *n.s.* | **-2.6 (1.0)** | **0.02** | **-2.4 (1.0)** | **0.03** |
|  |  | rD | 0.9 (0.5) | -0.2 (0.7) | 3.2 (0.8) | 0.7 (0.9) | *n.s.* | -0.9 (0.9) | *n.s.* | **2.2 (0.9)** | **0.02** | **3.7 (1.1)** | **0.002** |
|  |  | aD | 0.4 (0.3) | -0.2 (0.4) | 1.2 (0.4) | 0.2 (0.5) | *n.s.* | -0.6 (0.5) | *n.s.* | 0.9 (0.5) | *n.s.* | **1.4 (0.0)** | **0.01** |
| Middle occipital WM | Left | FA | -0.7 (0.7) | 0.4 (0.7) | -2.1 (0.5) | -1.0 (0.8) | *n.s.* | -0.0 (0.9) | *n.s.* | **-2.3 (0.8)** | **0.003** | -1.9 (0.9) | *n.s.* |
|  |  | rD | 0.8 (0.6) | 0.2 (0.7) | 3.3 (0.8) | 1.0 (0.9) | *n.s.* | -0.4 (0.9) | *n.s.* | **2.4 (0.9)** | **0.02** | **3.2 (1.1)** | **0.004** |
|  |  | aD | 0.6 (0.5) | -0.1 (0.4) | 1.4 (0.5) | 0.2 (0.6) | *n.s.* | -0.5 (0.6) | *n.s.* | 0.9 (0.7) | *n.s.* | **1.5 (0.6)** | **0.02** |
|  | Right | FA | -0.2 (0.5) | -0.2 (0.5) | -1.5 (0.5) | -0.9 (0.6) | *n.s.* | -0.2 (0.7) | *n.s.* | **-1.8 (0.7)** | **0.003** | -1.3 (0.7) | *n.s.* |
|  |  | rD | 0.7 (0.3) | -0.3 (0.5) | 2.6 (0.8) | 0.4 (0.7) | *n.s.* | -0.9 (0.6) | *n.s.* | **1.7 (0.8)** | **0.03** | **2.9 (0.9)** | **0.001** |
|  |  | aD | 0.6 (0.3) | -0.5 (0.3) | 1.3 (0.5) | -0.2 (0.5) | *n.s.* | **-1.0 (0.4)** | **0.02** | 0.7 (0.6) | *n.s.* | **1.7 (0.5)** | **0.001** |
| Inferior occipital WM | Left | FA | -1.0 (1.3) | -0.3 (0.7) | -1.8 (0.6) | -0.6 (0.9) | *n.s.* | 0.1 (1.2) | *n.s.* | -1.6 (1.2) | *n.s.* | -1.4 (0.9) | *n.s.* |
|  |  | rD | 0.7 (0.5) | -0.7 (0.7) | 1.8 (0.5) | 0.1 (0.8) | *n.s.* | -1.1 (0.9) | *n.s.* | 1.1 (0.7) | *n.s.* | **2.5 (0.9)** | **0.008** |
|  |  | aD | 0.5 (0.6) | -0.6 (0.6) | 1.0 (0.6) | -0.3 (0.7) | *n.s.* | -1.1 (0.8) | *n.s.* | 0.5 (0.8) | *n.s.* | 1.6 (0.8) | *n.s.* |
|  | Right | FA | -0.4 (0.5) | 0.0 (0.5) | -0.9 (0.6) | -0.9 (0.6) | *n.s.* | -0.5 (0.7) | *n.s.* | **-1.5 (0.8)** | **0.04** | -0.8 (0.8) | *n.s.* |
|  |  | rD | 0.7 (0.4) | -0.2 (0.6) | 1.7 (0.7) | 0.0 (0.7) | *n.s.* | -0.8 (0.7) | *n.s.* | 0.9 (0.8) | *n.s.* | 1.9 (0.9) | *n.s.* |
|  |  | aD | 0.9 (0.4) | -0.0 (0.5) | 1.2 (0.6) | -0.4 (0.6) | *n.s.* | -0.9 (0.6) | *n.s.* | 0.3 (0.7) | *n.s.* | 1.2 (0.7) | *n.s.* |
| Cuneus WM | Left | FA | -1.3 (1.4) | -0.5 (0.6) | -2.6 (0.8) | -1.4 (0.9) | *n.s.* | -0.3 (1.1) | *n.s.* | **-3.1 (1.3)** | **0.03** | **-2.1 (1.0)** | **0.04** |
|  |  | rD | 0.5 (0.6) | 0.1 (0.9) | 3.6 (1.2) | 1.4 (1.1) | *n.s.* | -0.1 (1.1) | *n.s.* | **2.7 (1.2)** | **0.04** | **3.7 (1.5)** | **0.03** |
|  |  | aD | 0.2 (0.5) | -0.2 (0.5) | 1.9 (0.7) | 0.6 (0.7) | *n.s.* | -0.4 (0.8) | *n.s.* | 1.6 (0.9) | *n.s.* | **2.1 (0.9)** | **0.03** |
|  | Right | FA | -0.9 (1.0) | 0.1 (0.7) | -2.6 (0.8) | -1.4 (1.0) | *n.s.* | 0.3 (1.1) | *n.s.* | **-3.8 (1.2)** | **0.001** | **-3.0 (1.0)** | **0.007** |
|  |  | rD | 0.5 (0.5) | -0.4 (0.6) | 3.6 (1.2) | 0.8 (1.0) | *n.s.* | -0.8 (0.8) | *n.s.* | 2.4 (1.1) | *n.s.* | **3.9 (1.3)** | **0.004** |
|  |  | aD | 0.5 (0.5) | -0.4 (0.5) | 1.8 (0.8) | 0.0 (0.7) | *n.s.* | -0.9 (0.7) | *n.s.* | 1.1 (0.9) | *n.s.* | **2.2 (0.9)** | **0.03** |
| Lingual WM | Left | FA | -0.8 (0.7) | -1.0 (0.6) | -2.2 (0.7) | -1.4 (0.8) | *n.s.* | -0.7 (0.9) | *n.s.* | **-2.4 (1.0)** | **0.03** | -1.4 (0.9) | *n.s.* |
|  |  | rD | 0.9 (0.7) | 2.8 (1.5) | 6.1 (1.7) | 3.3 (1.7) | *n.s.* | 2.1 (1.9) | *n.s.* | **4.4 (1.6)** | **0.02** | 2.3 (2.3) | *n.s.* |
|  |  | aD | 0.6 (0.6) | 1.8 (1.2) | 3.5 (1.0) | 2.1 (1.3) | *n.s.* | -1.4 (1.5) | *n.s.* | **2.9 (1.2)** | **0.03** | 1.3 (1.6) | *n.s.* |
|  | Right | FA | -0.4 (0.8) | 0.2 (0.8) | -2.3 (0.9) | -1.0 (1.0) | *n.s.* | 0.2 (1.1) | *n.s.* | **-2.7 (1.2)** | **0.03** | -2.4 (1.1) | *n.s.* |
|  |  | rD | 1.5 (0.6) | 1.4 (1.4) | 4.1 (1.6) | 1.0 (1.6) | *n.s.* | -0.1 (1.7) | *n.s.* | 2.1 (1.5) | *n.s.* | 2.4 (2.1) | *n.s.* |
|  |  | aD | 1.5 (0.6) | 1.1 (1.1) | 2.2 (1.1) | 0.2 (1.2) | *n.s.* | -0.3 (1.4) | *n.s.* | 0.7 (1.2) | *n.s.* | 1.0 (1.6) | *n.s.* |
| Fusiform WM | Left | FA | -1.5 (0.6) | 0.4 (0.6) | -2.1 (0.7) | 0.4 (0.8) | *n.s.* | 1.6 (0.8) | *n.s.* | -1.0 (0.9) | *n.s.* | **-2.5 (0.9)** | **0.01** |
|  |  | rD | 1.0 (0.6) | -0.2 (0.7) | 2.1 (0.5) | 0.1 (0.8) | *n.s.* | -1.2 (0.9) | *n.s.* | 1.3 (0.8) | *n.s.* | **1.5 (0.9)** | **0.008** |
|  |  | aD | 0.7 (0.6) | 0.1 (0.7) | 1.3 (0.5) | 0.1 (0.7) | *n.s.* | -0.6 (0.9) | *n.s.* | 0.7 (0.7) | *n.s.* | 1.3 (0.8) | *n.s.* |
|  | Right | FA | -0.4 (0.5) | 0.0 (0.6) | -1.4 (0.7) | -0.4 (0.7) | *n.s.* | 0.2 (0.8) | *n.s.* | -1.1 (0.8) | *n.s.* | -1.2 (0.9) | *n.s.* |
|  |  | rD | 0.6 (0.5) | -0.6 (0.7) | 2.0 (0.7) | 0.2 (0.9) | *n.s.* | -1.2 (0.9) | *n.s.* | 1.5 (0.9) | *n.s.* | **2.8 (1.0)** | **0.01** |
|  |  | aD | 0.5 (0.4) | -0.4 (0.6) | 1.5 (0.7) | 0.0 (0.7) | *n.s.* | -1.0 (0.7) | *n.s.* | 1.0 (0.7) | *n.s.* | **2.0 (0.9)** | **0.03** |
| Superior temporal WM | Left | FA | -0.5 (0.5) | -0.5 (0.5) | -1.7 (0.5) | -0.9 (0.6) | *n.s.* | -0.2 (0.7) | *n.s.* | **-1.7 (0.7)** | **0.01** | -1.4 (0.7) | *n.s.* |
|  |  | rD | 0.5 (0.3) | 0.1 (0.6) | 2.3 (0.5) | 0.7 (0.6) | *n.s.* | -0.3 (0.7) | *n.s.* | **1.7 (0.6)** | **0.005** | **2.2 (0.8)** | **0.008** |
|  |  | aD | 0.2 (0.3) | -0.2 (0.5) | 1.2 (0.4) | 0.3 (0.5) | *n.s.* | -0.4 (0.6) | *n.s.* | 1.0 (0.5) | *n.s.* | **1.4 (0.6)** | **0.03** |
|  | Right | FA | -0.5 (0.5) | -0.1 (0.5) | -1.1 (0.6) | -0.4 (0.7) | *n.s.* | 0.1 (0.7) | *n.s.* | -1.1 (0.8) | *n.s.* | -1.1 (0.8) | *n.s.* |
|  |  | rD | 0.8 (0.3) | 0.5 (0.6) | 2.4 (0.7) | 0.5 (0.7) | *n.s.* | -0.4 (0.6) | *n.s.* | 1.4 (0.7) | *n.s.* | **2.1 (0.9)** | **0.04** |
|  |  | aD | 0.7 (0.2) | 0.2 (0.4) | 1.5 (0.5) | 0.2 (0.5) | *n.s.* | -0.4 (0.4) | *n.s.* | 0.9 (0.5) | *n.s.* | **1.4 (0.6)** | **0.02** |
| Middle temporal WM | Left | FA | -0.7 (0.5) | -0.1 (0.5) | -1.9 (0.9) | -0.6 (0.6) | *n.s.* | 0.3 (0.7) | *n.s.* | **-1.7 (0.6)** | **0.01** | **-1.7 (0.7)** | **0.03** |
|  |  | rD | 0.6 (0.4) | -0.1 (0.6) | 2.2 (0.4) | 0.5 (0.6) | *n.s.* | -0.6 (0.7) | *n.s.* | **1.6 (0.5)** | **0.002** | **2.4 (0.7)** | **0.001** |
|  |  | aD | 0.2 (0.3) | -0.2 (0.4) | 1.2 (0.3) | 0.4 (0.5) | *n.s.* | -0.3 (0.5) | *n.s.* | **1.1 (0.5)** | **0.02** | **1.4 (0.5)** | **0.005** |
|  | Right | FA | -0.5 (0.4) | 0.3 (0.4) | -1.4 (0.6) | -0.2 (0.6) | *n.s.* | 0.6 (0.6) | *n.s.* | -1.1 (0.7) | *n.s.* | **-1.6 (0.7)** | **0.05** |
|  |  | rD | 1.0 (0.3) | -0.3 (0.5) | 2.4 (0.5) | 0.0 (0.6) | *n.s.* | -1.2 (0.5) | *n.s.* | 1.2 (0.6) | *n.s.* | **2.6 (0.7)** | **<0.001** |
|  |  | aD | 0.7 (0.3) | -0.2 (0.4) | 1.4 (0.5) | -0.1 (0.5) | *n.s.* | -0.9 (0.4) | *n.s.* | 0.8 (0.5) | *n.s.* | **1.7 (0.6)** | **0.001** |
| Inferior temporal WM | Left | FA | 0.1 (0.4) | 0.4 (0.4) | -2.7 (0.9) | -0.9 (0.7) | *n.s.* | 0.2 (0.6) | *n.s.* | **-2.6 (1.0)** | **0.01** | **-2.4 (0.8)** | **0.007** |
|  |  | rD | 0.8 (0.4) | 0.0 (0.7) | 2.0 (0.5) | 0.6 (0.7) | *n.s.* | -0.6 (0.8) | *n.s.* | **1.5 (0.6)** | **0.04** | **2.2 (0.9)** | **0.01** |
|  |  | aD | 0.7 (0.4) | 0.3 (0.6) | 1.3 (0.5) | 0.2 (0.6) | *n.s.* | -0.4 (0.7) | *n.s.* | 0.7 (0.6) | *n.s.* | 1.1 (0.7) | *n.s.* |
|  | Right | FA | -1.0 (0.5) | 0.2 (0.5) | -0.2 (2.5) | 0.7 (0.6) | *n.s.* | 0.9 (0.7) | *n.s.* | 0.5 (0.7) | *n.s.* | -0.3 (0.7) | *n.s.* |
|  |  | rD | 0.8 (0.4) | -0.2 (0.6) | 1.3 (0.5) | -0.1 (0.6) | *n.s.* | -0.9 (0.7) | *n.s.* | 0.6 (0.6) | *n.s.* | **1.6 (0.8)** | **0.04** |
|  |  | aD | 0.3 (0.3) | -0.1 (0.5) | 1.2 (0.5) | 0.3 (0.6) | *n.s.* | -0.4 (0.6) | *n.s.* | 0.9 (0.6) | *n.s.* | 1.4 (0.7) | *n.s.* |
| Caudal anterior CG | Left | FA | -0.5 (0.7) | -0.1 (0.7) | -1.0 (0.7) | -0.3 (0.8) | *n.s.* | 0.2 (0.9) | *n.s.* | -0.8 (0.9) | *n.s.* | -0.9 (1.0) | *n.s.* |
|  |  | rD | 0.6 (0.6) | 1.0 (0.9) | 2.7 (0.6) | 1.7 (1.0) | *n.s.* | 0.6 (1.2) | *n.s.* | **2.2 (0.8)** | **0.006** | 1.9 (1.1) | *n.s.* |
|  |  | aD | 0.0 (0.5) | 0.5 (0.7) | 1.6 (0.5) | 1.0 (0.7) | *n.s.* | 0.5 (0.9) | *n.s.* | **1.5 (0.7)** | **0.04** | 1.2 (0.9) | *n.s.* |
|  | Right | FA | 0.5 (0.9) | 0.1 (0.6) | -1.6 (0.8) | -1.3 (0.8) | *n.s.* | -0.5 (1.0) | *n.s.* | **-2.5 (1.1)** | **0.02** | -1.6 (1.0) | *n.s.* |
|  |  | rD | 0.5 (0.5) | 0.3 (0.9) | 3.9 (0.8) | 1.9 (1.0) | *n.s.* | -0.0 (1.1) | *n.s.* | **3.1 (0.8)** | **<0.001** | **4.1 (1.2)** | **0.001** |
|  |  | aD | 0.7 (0.5) | 0.3 (0.7) | 2.1 (0.6) | 0.5 (0.7) | *n.s.* | -0.4 (0.9) | *n.s.* | 1.4 (0.7) | *n.s.* | 1.8 (0.9) | *n.s.* |
| Mid-posterior cingulum | Left | FA | -0.4 (0.8) | -0.6 (0.9) | -0.2 (0.9) | -0.3 (1.0) | *n.s.* | -0.3 (1.2) | *n.s.* | -0.2 (1.1) | *n.s.* | 0.1 (1.3) | *n.s.* |
|  |  | rD | 0.1 (0.8) | 1.6 (1.3) | 3.0 (1.0) | 2.8 (1.4) | *n.s.* | 1.8 (1.6) | *n.s.* | **2.9 (1.2)** | **0.006** | 1.5 (1.6) | *n.s.* |
|  |  | aD | -0.4 (0.7) | 0.9 (0.7) | 2.1 (0.6) | **2.0 (0.8)** | **0.04** | 1.4 (1.0) | *n.s.* | **2.5 (0.9)** | **0.01** | 1.3 (0.9) | *n.s.* |
|  | Right | FA | 0.8 (1.0) | -0.2 (0.9) | -0.4 (1.0) | -1.2 (1.0) | *n.s.* | -1.1 (1.3) | *n.s.* | -1.4 (1.2) | *n.s.* | -0.3 (1.3) | *n.s.* |
|  |  | rD | -0.2 (0.8) | 0.7 (1.0) | 2.9 (0.8) | **2.7 (1.2)** | **0.04** | 1.3 (1.4) | *n.s.* | **3.3 (1.1)** | **0.008** | 2.5 (1.3) | *n.s.* |
|  |  | aD | 0.3 (0.6) | 0.5 (0.5) | 1.9 (0.5) | 1.0 (0.7) | *n.s.* | 0.2 (0.8) | *n.s.* | 1.7 (0.8) | *n.s.* | **1.6 (0.7)** | **0.03** |
| Isthmus cingulum | Left | FA | -0.4 (0.7) | 0.8 (0.8) | -0.9 (0.9) | 0.2 (0.9) | *n.s.* | 1.0 (1.0) | *n.s.* | -0.7 (1.1) | *n.s.* | -1.8 (1.2) | *n.s.* |
|  |  | rD | 0.3 (0.6) | -0.9 (0.9) | 2.7 (1.0) | 0.7 (1.1) | *n.s.* | -1.0 (1.1) | *n.s.* | 2.4 (1.1) | *n.s.* | **3.7 (1.3)** | **0.005** |
|  |  | aD | 0.1 (0.6) | -0.2 (0.7) | 1.9 (0.7) | 0.9 (0.8) | *n.s.* | -0.1 (1.0) | *n.s.* | 1.8 (0.9) | *n.s.* | 2.1 (1.0) | *n.s.* |
|  | Right | FA | 0.1 (1.0) | 0.4 (0.8) | -0.7 (0.9) | -0.5 (1.0) | *n.s.* | 0.2 (1.1) | *n.s.* | -1.2 (1.3) | *n.s.* | -1.2 (1.1) | *n.s.* |
|  |  | rD | 0.2 (0.5) | -0.6 (0.7) | 2.2 (1.0) | 0.7 (0.9) | *n.s.* | -0.6 (0.9) | *n.s.* | 1.9 (1.1) | *n.s.* | **2.9 (1.2)** | **0.02** |
|  |  | aD | 0.3 (0.5) | -0.3 (0.6) | 1.7 (0.8) | 0.5 (0.8) | *n.s.* | -0.5 (0.8) | *n.s.* | 1.5 (0.9) | *n.s.* | 2.0 (1.0) | *n.s.* |
| Para-hippocampal cingulum | Left | FA | -0.9 (0.5) | 1.0 (0.7) | -2.2 (0.9) | 0.2 (0.9) | *n.s.* | 1.8 (0.9) | *n.s.* | -1.6 (1.1) | *n.s.* | **-3.2 (1.1)** | **0.007** |
|  |  | rD | 1.0 (0.3) | -0.4 (0.7) | 4.1 (1.1) | 0.6 (1.0) | *n.s.* | -1.4 (0.7) | *n.s.* | **2.6 (1.0)** | **0.02** | **4.7 (1.3)** | **0.001** |
|  |  | aD | 0.5 (0.3) | 0.2 (0.4) | 2.4 (0.7) | 0.8 (0.6) | *n.s.* | -0.2 (0.5) | *n.s.* | **1.8 (0.7)** | **0.01** | **2.2 (0.8)** | **0.007** |
|  | Right | FA | 0.1 (0.6) | 0.3 (0.7) | -2.3 (0.9) | -1.1 (0.9) | *n.s.* | 0.2 (0.9) | *n.s.* | **-2.7 (1.1)** | **0.03** | -2.6 (1.1) | *n.s.* |
|  |  | rD | 0.5 (0.5) | -0.1 (0.8) | 3.8 (1.5) | 1.4 (1.2) | *n.s.* | -0.4 (1.0) | *n.s.* | 3.2 (1.5) | *n.s.* | **4.1 (1.7)** | **0.03** |
|  |  | aD | 0.4 (0.4) | -0.1 (0.5) | 2.0 (0.9) | 0.5 (0.8) | *n.s.* | -0.5 (0.7) | *n.s.* | 1.6 (1.0) | *n.s.* | **2.1 (1.0)** | **0.04** |
| Genu of callosum | — | FA | -0.2 (0.5) | -0.0 (0.6) | -1.9 (0.6) | -0.8 (0.6) | *n.s.* | -0.0 (0.7) | *n.s.* | -1.8 (0.7) | *n.s.* | -1.7 (0.8) | *n.s.* |
|  |  | rD | 1.4 (0.6) | 1.2 (1.4) | 4.2 (1.0) | 1.6 (1.4) | *n.s.* | -0.5 (1.5) | *n.s.* | 2.8 (1.1) | *n.s.* | **4.1 (1.6)** | **0.02** |
|  |  | aD | 1.3 (0.5) | 0.7 (0.5) | 1.6 (0.5) | -0.1 (0.6) | *n.s.* | 0.6 (0.7) | *n.s.* | 0.4 (0.7) | *n.s.* | 0.9 (0.7) | *n.s.* |
| Superior longitudinal fasciculus | Left | FA | -0.2 (1.0) | -0.5 (0.6) | -1.3 (0.5) | -1.1 (0.7) | *n.s.* | -0.8 (1.0) | *n.s.* | -1.8 (0.9) | *n.s.* | -0.8 (0.8) | *n.s.* |
|  |  | rD | 0.1 (0.5) | 0.5 (0.7) | 2.5 (0.6) | 1.6 (0.8) | *n.s.* | 0.6 (0.9) | *n.s.* | **2.3 (0.7)** | **0.005** | **2.0 (0.9)** | **0.04** |
|  |  | aD | -0.2 (0.5) | 0.1 (0.4) | 1.3 (0.5) | 1.0 (0.6) | *n.s.* | 0.4 (0.6) | *n.s.* | **1.5 (0.7)** | **0.02** | **1.2 (0.6)** | **0.04** |
|  | Right | FA | -1.4 (0.9) | -0.7 (0.6) | -1.1 (0.5) | -0.5 (0.7) | *n.s.* | -0.2 (0.9) | *n.s.* | -1.0 (0.9) | *n.s.* | -0.6 (0.8) | *n.s.* |
|  |  | rD | 0.6 (0.3) | 0.5 (0.5) | 2.7 (0.7) | 1.0 (0.7) | *n.s.* | 0.0 (0.6) | *n.s.* | **1.8 (0.7)** | **0.01** | **2.1 (0.8)** | **0.02** |
|  |  | aD | 0.1 (0.4) | 0.1 (0.4) | 1.4 (0.5) | 0.6 (0.5) | *n.s.* | -0.0 (0.5) | *n.s.* | **1.3 (0.6)** | **0.03** | **1.4 (0.6)** | **0.03** |
| Sagittal stratum & Inferior longitudinal fasciculus | Left | FA | -0.9 (0.5) | 0.1 (0.4) | -2.0 (0.5) | -0.4 (0.5) | *n.s.* | 0.5 (0.6) | *n.s.* | **-1.6 (0.7)** | **0.02** | **-1.9 (0.6)** | **0.002** |
|  |  | rD | 1.7 (0.5) | 0.5 (1.2) | 4.5 (1.0) | 1.1 (1.2) | *n.s.* | -1.4 (1.2) | *n.s.* | **2.8 (1.0)** | **0.008** | **5.0 (1.4)** | **0.001** |
|  |  | aD | 1.0 (0.5) | 0.3 (0.6) | 2.2 (0.7) | 0.4 (0.7) | *n.s.* | -0.7 (0.7) | *n.s.* | 1.4 (0.8) | *n.s.* | **2.1 (0.9)** | **0.01** |
|  | Right | FA | -0.4 (0.5) | -0.2 (0.6) | -2.1 (0.8) | -0.7 (0.7) | *n.s.* | 0.0 (0.8) | *n.s.* | -1.7 (0.8) | *n.s.* | -1.7 (0.9) | *n.s.* |
|  |  | rD | 1.6 (0.7) | 1.8 (1.4) | 4.6 (1.3) | 2.3 (1.6) | *n.s.* | -0.0 (1.6) | *n.s.* | **3.7 (1.6)** | **0.03** | **4.5 (1.9)** | **0.03** |
|  |  | aD | 1.1 (0.4) | 1.1 (0.7) | 2.8 (1.0) | 1.0 (0.9) | *n.s.* | 0.0 (0.8) | *n.s.* | 1.9 (1.1) | *n.s.* | 2.1 (1.2) | *n.s.* |
| Superior fronto-occipital fasciculus | Left | FA | -1.0 (1.0) | -2.6 (0.9) | -3.9 (0.9) | **-2.2 (1.0)** | **0.01** | -1.4 (1.2) | *n.s.* | **-3.2 (1.2)** | **0.001** | -2.0 (1.2) | *n.s.* |
|  |  | rD | 1.7 (1.4) | 6.2 (1.6) | 13.1 (1.9) | **10.5 (2.6)** | **0.005** | 6.4 (2.8) | *n.s.* | **10.3 (2.0)** | **<0.001** | 6.1 (2.4) | *n.s.* |
|  |  | aD | 0.9 (1.1) | 3.6 (1.4) | 6.4 (1.4) | 5.2 (1.8) | *n.s.* | 3.3 (2.1) | *n.s.* | 5.4 (1.6) | *n.s.* | 3.5 (1.9) | *n.s.* |
|  | Right | FA | -2.4 (1.2) | -1.7 (1.0) | -4.0 (0.8) | -0.6 (1.1) | *n.s.* | 0.8 (1.4) | *n.s.* | -2.1 (1.3) | *n.s.* | **-3.2 (1.3)** | **0.02** |
|  |  | rD | 1.2 (0.9) | 5.8 (2.2) | 12.8 (2.4) | **10.6 (3.1)** | **0.003** | 4.5 (2.6) | *n.s.* | **11.0 (2.3)** | **<0.001** | **11.4 (3.2)** | **<0.001** |
|  |  | aD | 0.1 (0.7) | 3.3 (1.6) | 9.1 (2.1) | **7.5 (2.3)** | **0.008** | 3.4 (1.8) | *n.s.* | **8.1 (2.0)** | **<0.001** | **8.4 (2.7)** | **0.002** |
| Posterior thalamic radiation | Left | FA | -1.1 (0.5) | -0.5 (0.6) | -1.9 (0.4) | -0.6 (0.6) | *n.s.* | 0.2 (0.8) | *n.s.* | **-1.6 (0.6)** | **0.03** | -1.5 (0.7) | *n.s.* |
|  |  | rD | 1.4 (0.6) | 2.2 (1.4) | 7.8 (1.7) | 2.5 (1.6) | *n.s.* | 0.5 (1.5) | *n.s.* | **0.0 (1.3)** | **0.04** | 4.4 (2.2) | *n.s.* |
|  |  | aD | 0.5 (0.5) | 0.8 (0.6) | 2.3 (0.7) | 0.0 (0.7) | *n.s.* | 0.4 (0.7) | *n.s.* | 1.7 (0.8) | *n.s.* | 1.3 (0.8) | *n.s.* |
|  | Right | FA | -0.8 (0.6) | -0.7 (0.5) | -1.8 (0.6) | -1.0 (0.7) | *n.s.* | -0.5 (0.8) | *n.s.* | -1.8 (0.8) | *n.s.* | -1.1 (0.8) | *n.s.* |
|  |  | rD | 1.3 (0.6) | 2.3 (1.1) | 6.9 (1.9) | 2.7 (1.5) | *n.s.* | 0.8 (1.3) | *n.s.* | 4.0 (1.6) | *n.s.* | 4.1 (2.1) | *n.s.* |
|  |  | aD | 0.9 (0.5) | 0.9 (0.5) | 2.6 (0.9) | 0.8 (0.7) | *n.s.* | 0.0 (0.7) | *n.s.* | 1.7 (0.9) | *n.s.* | 1.6 (0.9) | *n.s.* |
| Posterior corona radiata | Left | FA | -0.4 (0.5) | 0.7 (0.7) | -0.3 (0.5) | 0.3 (0.7) | *n.s.* | 1.1 (0.9) | *n.s.* | -0.4 (0.7) | *n.s.* | -1.3 (0.8) | *n.s.* |
|  |  | rD | 0.4 (0.6) | 0.6 (0.8) | 5.7 (1.5) | 2.2 (1.1) | *n.s.* | 0.3 (1.0) | *n.s.* | 4.0 (1.2) | *n.s.* | **4.3 (1.4)** | **0.03** |
|  |  | aD | 0.1 (0.5) | 1.3 (0.5) | 3.6 (0.8) | **2.5 (0.8)** | **0.04** | 1.3 (0.7) | *n.s.* | **3.4 (0.9)** | **0.02** | **2.5 (0.8)** | **0.009** |
|  | Right | FA | -0.9 (0.8) | -0.2 (0.5) | -1.0 (0.5) | -0.3 (0.7) | *n.s.* | 0.3 (0.8) | *n.s.* | -1.0 (0.9) | *n.s.* | -1.0 (0.7) | *n.s.* |
|  |  | rD | 0.9 (0.5) | 1.3 (0.7) | 5.9 (1.4) | 2.2 (2.1) | *n.s.* | 0.3 (0.8) | *n.s.* | 3.7 (1.2) | *n.s.* | **4.7 (1.4)** | **0.02** |
|  |  | aD | 0.6 (0.5) | 1.2 (0.6) | 3.9 (0.9) | 2.1 (0.9) | *n.s.* | 0.6 (0.8) | *n.s.* | 3.3 (1.0) | *n.s.* | **3.1 (1.0)** | **0.01** |
| Retro-lenticular part of internal capsule | Left | FA | -0.4 (0.5) | 0.1 (0.6) | -1.4 (0.5) | -0.5 (0.7) | *n.s.* | 0.4 (0.8) | *n.s.* | -1.4 (0.7) | *n.s.* | **-1.8 (0.8)** | **0.04** |
|  |  | rD | 0.6 (0.6) | 0.3 (0.9) | 3.4 (1.2) | 0.9 (1.1) | *n.s.* | -0.3 (1.1) | *n.s.* | **1.2 (1.1)** | **0.04** | 2.7 (1.4) | *n.s.* |
|  |  | aD | 0.2 (0.4) | 0.5 (0.5) | 0.8 (0.5) | 0.5 (0.6) | *n.s.* | 0.3 (0.7) | *n.s.* | 0.7 (0.7) | *n.s.* | 0.3 (0.7) | *n.s.* |
|  | Right | FA | -0.3 (0.6) | -0.4 (0.7) | -1.1 (0.6) | -0.6 (0.8) | *n.s.* | -0.1 (0.9) | *n.s.* | -1.1 (0.8) | *n.s.* | -1.1 (0.9) | *n.s.* |
|  |  | rD | 0.7 (0.8) | 0.9 (0.8) | 5.2 (1.8) | 1.8 (1.3) | *n.s.* | 0.3 (1.2) | *n.s.* | 3.6 (1.7) | *n.s.* | 3.1 (1.6) | *n.s.* |
|  |  | aD | 0.3 (0.5) | 0.3 (0.5) | 2.1 (0.7) | 0.9 (0.7) | *n.s.* | 0.1 (0.7) | *n.s.* | 1.8 (0.9) | *n.s.* | 1.8 (0.9) | *n.s.* |
| Anterior limb of internal capsule | Left | FA | -1.1 (0.8) | -1.3 (0.7) | -1.2 (0.7) | -0.8 (0.8) | *n.s.* | -0.7 (1.0) | *n.s.* | -1.0 (1.0) | *n.s.* | -0.3 (1.0) | *n.s.* |
|  |  | rD | 0.4 (0.7) | 1.1 (0.8) | 3.7 (1.1) | 2.0 (1.1) | *n.s.* | 0.9 (1.1) | *n.s.* | **2.8 (1.2)** | **0.02** | 1.9 (1.3) | *n.s.* |
|  |  | aD | -0.1 (0.6) | -0.1 (0.6) | 1.3 (0.6) | 0.9 (0.8) | *n.s.* | 0.1 (0.9) | *n.s.* | 1.6 (0.8) | *n.s.* | 1.5 (0.8) | *n.s.* |
|  | Right | FA | -0.6 (0.8) | -0.9 (0.7) | -1.7 (0.5) | -1.1 (0.8) | *n.s.* | -0.6 (1.0) | *n.s.* | -1.9 (0.9) | *n.s.* | -1.1 (0.9) | *n.s.* |
|  |  | rD | 0.6 (0.6) | 1.2 (0.7) | 4.8 (1.2) | 2.0 (1.0) | *n.s.* | 0.6 (1.0) | *n.s.* | **3.2 (1.2)** | **0.02** | 2.8 (1.3) | *n.s.* |
|  |  | aD | 0.4 (0.4) | 0.0 (0.5) | 1.4 (0.7) | 0.3 (0.7) | *n.s.* | -0.4 (0.7) | *n.s.* | 1.1 (0.8) | *n.s.* | 1.4 (0.8) | *n.s.* |
| External capsule | Left | FA | -0.8 (0.6) | -0.7 (0.6) | -0.1 (0.7) | -0.1 (0.8) | *n.s.* | -0.4 (0.9) | *n.s.* | 0.2 (0.9) | *n.s.* | 0.5 (0.9) | *n.s.* |
|  |  | rD | 0.2 (0.4) | 0.5 (0.6) | 0.5 (0.7) | 0.5 (0.7) | *n.s.* | 0.5 (0.7) | *n.s.* | 0.5 (0.7) | *n.s.* | -0.1 (0.9) | *n.s.* |
|  |  | aD | 0.1 (0.4) | 0.1 (0.4) | 0.2 (0.5) | 0.1 (0.5) | *n.s.* | 0.1 (0.6) | *n.s.* | 0.2 (0.6) | *n.s.* | 0.1 (0.6) | *n.s.* |
|  | Right | FA | -1.2 (0.7) | 0.2 (0.6) | -0.3 (0.6) | 0.6 (0.7) | *n.s.* | 1.0 (0.9) | *n.s.* | 0.2 (0.9) | *n.s.* | -0.6 (0.8) | *n.s.* |
|  |  | rD | 0.4 (0.4) | 0.2 (0.5) | 1.8 (0.5) | 0.7 (0.6) | *n.s.* | -0.1 (0.7) | *n.s.* | 1.4 (0.7) | *n.s.* | 1.6 (0.8) | *n.s.* |
|  |  | aD | 0.1 (0.3) | 0.3 (0.4) | 1.4 (0.5) | 0.8 (0.5) | *n.s.* | 0.3 (0.5) | *n.s.* | **1.4 (0.6)** | **0.03** | 1.1 (0.6) | *n.s.* |
| Cortico-spinal tract (in brain stem) | Left | FA | -2.0 (1.1) | -1.6 (1.2) | -1.4 (1.0) | -0.2 (1.3) | *n.s.* | -0.0 (1.6) | *n.s.* | -0.5 (1.5) | *n.s.* | -0.5 (1.6) | *n.s.* |
|  |  | rD | 1.0 (1.2) | -0.7 (2.0) | 5.1 (2.5) | 0.2 (2.2) | *n.s.* | -1.7 (2.3) | *n.s.* | 2.7 (2.4) | *n.s.* | 5.1 (3.2) | *n.s.* |
|  |  | aD | -0.1 (0.9) | -1.8 (1.5) | 2.7 (1.2) | 0.3 (1.6) | *n.s.* | -1.7 (1.8) | *n.s.* | 2.6 (1.5) | *n.s.* | 4.6 (2.1) | *n.s.* |
|  | Right | FA | -1.7 (1.3) | -0.8 (1.1) | -1.9 (0.9) | -0.4 (1.3) | *n.s.* | 0.3 (1.6) | *n.s.* | -1.2 (1.5) | *n.s.* | -1.2 (1.5) | *n.s.* |
|  |  | rD | 1.2 (0.9) | -0.8 (1.3) | 5.0 (1.9) | 0.3 (1.7) | *n.s.* | -1.9 (1.6) | *n.s.* | 2.9 (2.0) | *n.s.* | **5.7 (2.2)** | **0.02** |
|  |  | aD | 0.5 (0.8) | -1.5 (1.0) | 3.1 (1.3) | -0.0 (1.3) | *n.s.* | -2.1 (1.3) | *n.s.* | 2.4 (1.5) | *n.s.* | **4.7 (1.6)** | **0.008** |
| Medial lemniscus | Left | FA | -2.0 (1.0) | -2.4 (1.2) | -0.5 (1.1) | -0.0 (1.2) | *n.s.* | -1.0 (1.5) | *n.s.* | 1.0 (1.4) | *n.s.* | 1.8 (1.6) | *n.s.* |
|  |  | rD | -0.7 (1.2) | 1.8 (1.8) | 0.5 (2.4) | 2.1 (2.2) | *n.s.* | 2.6 (2.3) | *n.s.* | 1.5 (2.5) | *n.s.* | -1.5 (2.9) | *n.s.* |
|  |  | aD | -1.6 (1.0) | -0.3 (1.1) | 0.4 (1.5) | 1.6 (1.4) | *n.s.* | 1.1 (1.5) | *n.s.* | 2.4 (1.8) | *n.s.* | 0.9 (1.8) | *n.s.* |
|  | Right | FA | -1.3 (1.2) | -1.3 (1.0) | -1.0 (1.2) | -0.5 (1.3) | *n.s.* | -0.8 (1.5) | *n.s.* | -0.3 (1.7) | *n.s.* | 0.6 (1.5) | *n.s.* |
|  |  | rD | -1.6 (1.3) | 2.6 (1.8) | 1.4 (2.5) | 4.2 (1.1) | *n.s.* | 4.3 (2.1) | *n.s.* | 3.5 (2.6) | *n.s.* | -1.6 (3.0) | *n.s.* |
|  |  | aD | -2.1 (0.9) | 0.8 (1.1) | 0.6 (1.7) | **2.0 (1.4)** | **0.04** | 2.8 (1.4) | *n.s.* | 3.3 (1.8) | *n.s.* | -0.1 (1.8) | *n.s.* |
| Superior cerebellar peduncle | Left | FA | -0.3 (0.5) | -2.6 (0.8) | -0.6 (0.7) | -1.1 (0.7) | *n.s.* | **-1.9 (0.8)** | **0.01** | -0.5 (0.8) | *n.s.* | 1.1 (1.0) | *n.s.* |
|  |  | rD | 0.2 (0.6) | 1.9 (0.7) | 3.7 (1.4) | **2.8 (1.2)** | **0.01** | 2.0 (1.0) | *n.s.* | **3.1 (1.3)** | **0.01** | 0.6 (1.4) | *n.s.* |
|  |  | aD | 0.2 (0.5) | 0.6 (0.5) | 2.3 (1.1) | 1.3 (0.9) | *n.s.* | 0.5 (0.8) | *n.s.* | 2.1 (1.1) | *n.s.* | 1.6 (1.1) | *n.s.* |
|  | Right | FA | 0.3 (0.5) | -2.7 (1.1) | -0.2 (0.6) | -1.5 (0.8) | *n.s.* | **-2.6 (1.2)** | **0.02** | -0.8 (0.8) | *n.s.* | 1.4 (1.1) | *n.s.* |
|  |  | rD | -0.2 (0.6) | 2.0 (0.8) | 4.8 (2.8) | **3.7 (1.2)** | **0.008** | **2.5 (1.2)** | **0.02** | **4.1 (1.8)** | **0.04** | 0.4 (1.9) | *n.s.* |
|  |  | aD | -0.0 (0.5) | 0.6 (0.5) | 2.7 (1.5) | 1.7 (0.9) | *n.s.* | 0.7 (0.7) | *n.s.* | 2.8 (1.3) | *n.s.* | 1.9 (1.2) | *n.s.* |
| Midbrain | Left | FA | 0.3 (1.3) | -3.3 (1.8) | -0.5 (0.8) | **-2.3 (1.5)** | **0.03** | **-3.6 (2.3)** | **0.04** | -1.1 (1.4) | *n.s.* | 1.5 (1.8) | *n.s.* |
|  |  | rD | 0.1 (0.9) | 3.4 (1.1) | 2.7 (1.3) | **3.5 (1.4)** | **0.003** | **3.8 (1.5)** | **0.004** | **2.5 (1.4)** | **0.03** | -1.9 (1.6) | *n.s.* |
|  |  | aD | 0.1 (0.6) | 2.5 (0.8) | 1.9 (0.9) | **2.5 (1.0)** | **0.01** | **2.7 (1.1)** | **0.01** | 1.8 (1.0) | *n.s.* | -1.2 (1.2) | *n.s.* |
|  | Right | FA | 0.3 (1.5) | -2.4 (1.7) | -0.2 (0.8) | -1.8 (1.6) | *n.s.* | -2.8 (2.4) | *n.s.* | -0.8 (1.5) | *n.s.* | 1.4 (1.9) | *n.s.* |
|  |  | rD | 0.0 (1.0) | 3.1 (1.1) | 3.2 (1.3) | **3.6 (1.5)** | **0.01** | **3.6 (1.6)** | **0.04** | 3.1 (1.6) | *n.s.* | -0.9 (1.7) | *n.s.* |
|  |  | aD | -0.1 (0.7) | 2.2 (0.7) | 3.1 (1.0) | **3.1 (1.1)** | **0.005** | **2.7 (1.1)** | **0.02** | **3.3 (1.2)** | **0.03** | 0.2 (1.2) | *n.s.* |
| Caudate nucleus | Left | FA | -2.8 (1.4) | -2.0 (0.8) | -3.7 (1.0) | -0.8 (1.1) | *n.s.* | 0.1 (1.3) | *n.s.* | -1.9 (1.4) | *n.s.* | -1.9 (1.2) | *n.s.* |
|  |  | rD | 3.2 (1.2) | 9.0 (2.0) | 14.4 (2.6) | **21.2 (5.2)** | **<0.001** | **15.8 (5.3)** | **0.004** | **13.1 (2.7)** | **<0.001** | 4.9 (3.0) | *n.s.* |
|  |  | aD | 1.6 (0.9) | 6.7 (1.6) | 10.7 (2.1) | **13.8 (3.3)** | **<0.001** | **10.7 (3.4)** | **0.002** | **10.4 (2.2)** | **<0.001** | 3.6 (2.4) | *n.s.* |
|  | Right | FA | -3.0 (1.4) | -2.3 (0.6) | -4.4 (0.7) | -1.1 (1.0) | *n.s.* | -0.2 (1.2) | *n.s.* | -2.2 (1.4) | *n.s.* | -1.7 (0.9) | *n.s.* |
|  |  | rD | 5.5 (1.7) | 12.0 (2.1) | 13.8 (2.2) | **24.9 (5.8)** | **<0.001** | **17.6 (5.7)** | **0.003** | **13.1 (2.6)** | **<0.001** | **8.4 (3.0)** | **0.006** |
|  |  | aD | 3.9 (1.3) | 8.1 (1.5) | 11.0 (1.8) | **14.7 (3.5)** | **<0.001** | **9.6 (3.2)** | **0.004** | **10.6 (2.1)** | **0.001** | **6.7 (2.3)** | **0.005** |
| Thalamus | Left | FA | -1.2 (0.6) | -1.3 (0.5) | -1.0 (0.5) | -0.4 (0.6) | *n.s.* | -0.1 (0.8) | *n.s.* | -0.6 (0.7) | *n.s.* | -0.5 (0.7) | *n.s.* |
|  |  | rD | 1.0 (0.8) | 4.1 (1.0) | 11.3 (2.9) | **6.5 (1.8)** | **<0.001** | **4.5 (1.6)** | **0.005** | **7.4 (2.1)** | **0.001** | 1.4 (1.8) | *n.s.* |
|  |  | aD | 0.3 (0.6) | 2.9 (0.8) | 5.6 (1.4) | **4.4 (1.2)** | **<0.001** | **3.4 (1.1)** | **0.004** | **5.2 (1.3)** | **<0.001** | 1.3 (1.3) | *n.s.* |
|  | Right | FA | -0.5 (0.6) | -1.9 (0.6) | -1.5 (0.5) | **-1.5 (0.6)** | **0.03** | -1.4 (0.8) | *n.s.* | -1.5 (0.7) | *n.s.* | -0.3 (0.8) | *n.s.* |
|  |  | rD | 2.1 (1.0) | 5.0 (1.1) | 7.6 (2.3) | **5.5 (2.0)** | **0.01** | **4.6 (1.8)** | **0.02** | **4.9 (2.1)** | **0.04** | 0.2 (1.9) | *n.s.* |
|  |  | aD | 1.4 (0.7) | 3.2 (0.8) | 3.8 (1.1) | **2.8 (1.2)** | **0.03** | 2.6 (1.2) | *n.s.* | 2.7 (1.2) | *n.s.* | 0.2 (1.2) | *n.s.* |

FA = fractional anisotropy; rD = radial diffusivity; aD = axial diffusivity; WM: white matter;

— : left and right regions were combined;

*n.s.: P_FDR_* ≥ 0.05

*Regions had no significant observations of any DTI variable in comparison of rates between groups are not listed.
